# Supplementary material for: Does use of GP and specialist services vary across areas and according to individual socioeconomic position? A multilevel analysis using linked data in Australia
Source: BMJ Open. 2024 Jan 6;14(1):e074624. doi: 10.1136/bmjopen-2023-074624 (PMC10773367; doi:10.1136/bmjopen-2023-074624)
Supplement: Supplementary data [file bmjopen-2023-074624supp001.pdf]

Title: Does use of GP and specialist services vary across areas and according to individual-socioeconomic position? A multilevel analysis using linked data in Australia.

Butler DC, Larkins S, Jorm L, et al *BMJ Open* 2023;:1–9. doi: bmjopen-2023-074624

Supplementary table 1: Item numbers for GP services

MBS item groups and name of group	Specific item numbers within item group	Notes on item numbers
A1-GP attendances	3, 23, 36, 44<br><br>Remainder A1 attendances=4, 20, 24, 35, 37, 43, 47, 51	Items for services in consulting rooms<br>Items relate to consultations at residential care OR other than residential care/consulting rooms
A2-MPs other than GP	52,53,54,57<br><br>Remainder of A2 attendances=58–60, 65, 92, 93, 95, 96	Items for services in consulting rooms<br>Items relate to consultations at residential care OR other than residential care/consulting rooms
A11-GP	597, 599	Urgent after-hours care
A11-MP other than GP	598, 600	Urgent after-hours care
A14-heath Assessments	701, 703, 705, 707	
A14-health assessments for Aboriginal and Torres Strait Islander peoples	715	
A15-GP management plans	721, 723, 729, 731, 732	Care plans, care plan reviews and team care arrangements
A15-case conference	735, 739, 743, 747, 750, 758	
A17	900 and 903	Medication reviews
A18-GP, cervical screening	2497, 2501, 2503, 2504, 2506, 2507, 2509	Incentive for overdue pap smears (>4 years since last pap)
A18-GP asthma	2546, 2547, 2552, 2553, 2558, 2559	cycle of care
A18-GP diabetes	2517, 2518, 2521, 2522, 2525, 2526	cycle of care
A19-non-referred cervical screening	2598, 2600, 2603, 2606, 2610, 2613, 2616	As per A19 for non-VR MPs
A19-non-referred, asthma	2664, 2666, 2668, 2673, 2675, 2677	As for A18 for non-VR MPs
A19-non-referred, diabetes	2620, 2622, 2624, 2631, 2633, 2635	As for A18 for non-VR MPs
A20-GP mental health care plans	2700, 2701, 2712, 2713, 2715, 2717, 2721, 2723, 2725, 2727	Preparation and review of mental health care plans
A22-after hours (GP attendances)	5000, 5003, 5010, 5020, 5023, 5028, 5040, 5043, 5049, 5060, 5063, 5067	Includes consultation room, residential facility and other. At consultation rooms bolded
A23-after hours (non-referred)	5200, 5203, 5207, 5208, 5220, 5223, 5227, 5228, 5260, 5263, 5265, 5267	As per A22 for non-VR MPs

Notes:

1. GP, general practitioner; MP, medical practitioner; VR vocationally registered.
2. All item numbers were included for frequency of GP use and continuity of care outcome measures. The specific item numbers included for length of consultation were 36, 44, 54, and 57. The specific item numbers for care planning included all those listed under A15 GP management plans.
3. Medical practitioners (MPs) in this case refer to medical doctors who provide non-referred services but are not vocationally registered as a GP (i.e. have not undertaken specialist training in General Practice or other equivalent recognised training program).

Supplementary table 2. Item numbers included for specialist use

MBS item groups and name of group	Specific item numbers within item group	Notes on item numbers
A3-specialist	99, 104, 105, 106, 107, 108, 109, 113	includes home visits; visits at surgery or hospital 99; 113=telehealth
A4-consultant physician	110, 112, 114, 116, 119, 122, 128, 131, 132, 133	112 & 114 telehealth; 122=home visit
A28-geriatric medicine	141, 143,145, 147, 149	145=home visit; 149 telehealth
A8-consultant psychiatry	288, 291, 293, 296, 299, 300, 302, 304, 306, 308, 310, 312, 314, 316, 318, 319, 330, 332, 334, 336, 338, 342, 344, 346, 353, 355, 356, 357, 358, 359, 361, 364, 366, 367, 369, 370	288=telehealth; 289 not included, for autism developmental delay, referred from any practitioner; 299=home visit, hospital consults excluded, interviewing other than patient excluded; 353–370 telepsychiatry
A12-occupational physician	384, 385, 386, 387, 388, 389	
A13-public health	410, 411, 412, 413, 414, 415, 416, 417	Case conferences excluded
A24-pain and palliative medicine	2799, 2801, 2806, 2814, 2820, 2824, 2832, 2840, 3003, 3005, 3010, 3014, 3015, 3018, 3023, 3028	Case conferences excluded
A26-neurosurgery	6004, 6007, 6009, 6011, 6013, 6015, 6016	

1. Note:
2. A3-specialist also includes all attendances by surgeons (at surgery/consulting rooms or hospital). With respect to surgical specialists, only neurosurgery has specific item numbers for attendances.

Supplementary table 3. Between-area variation in odds of above-average GP use.

	Null model	Model 2
**Cities**		
ICC	0.044	0.028
PCV (%)	na	36
MOR	1.45	1.34
**Inner regional**		
ICC	0.023	0.026
PCV (%)	na	-13
MOR	1.30	1.32
**Outer regional/remote**		
ICC	0.022	0.029
PCV (%)	na	-32
MOR	1.29	1.35

Notes:

1. ICC, intra-class correlation coefficient; PCV, proportional change in variance (reported change from null model); MOR, median odds ratio.
2. Null model, random intercept only, no covariates; Model 2 adjusted for education, age, sex, country of birth, marital status, self-rated health, chronic disease and physical functional limitation.
3. Wald test that between-area variance for the null model is non zero (i.e. δ_u^2 =0) p<.001. See Appendix G for full details of model diagnostics.

Supplementary table 4. Between-area variation in odds of quality of GP care outcomes

	Long consults		Continuity of care		Care planning	
	**Null model**	**Model 2**	**Null model**	**Model 2**	**Null model**	**Model 2**
**Cities**						
ICC	0.009	0.013	0.007	0.007	0.049	0.039
PCV (%)	na	-44	na	1.5	na	22
MOR	1.18	1.21	1.16	1.16	1.48	1.41
**Inner regional**						
ICC	0.027	0.031	0.017	0.019	0.049	0.048
PCV (%)	na	-16	na	-8	na	4
MOR	1.33	1.36	1.26	1.27	1.48	1.47
**Outer regional**						
ICC	0.033	0.041	0.037	0.037	0.043	0.044
PCV (%)	na	-24	na	-0.4	na	-2.1
MOR	1.37	1.43	1.40	1.40	1.44	1.45

Notes:

1. ICC: intra-class correlation coefficient; PCV: proportional change in variance (reported change from null model), MOR median odds ratio; na, not applicable.
2. Null model, random intercept only, no covariates;Model 2 adjusted for education, age, sex, country of birth, marital status, self-rated health, chronic disease and physical functional limitation.
3. Wald test that between-area variance for the null model is non zero (i.e. δ_u^2 =0) p<.001 for all outcomes.

Supplementary table 5. Between-area variation in the odds of specialist use

	Null model	Model 2
**Major cities**		
ICC	0.007	0.007
PCV (%)	na	-8
MOR	1.15	1.16
**Inner regional**		
ICC	0.008	0.009
PCV (%)	na	-19
MOR	1.17	1.18
**Outer regional**		
ICC	0.008	0.008
PCV (%)	na	-8
MOR	1.16	1.17

Notes:

1. ICC: intra-class correlation coefficient; PCV: proportional change in variance. Reported change from null model; MOR, median odds ratio; na, not applicable.
2. Null model, random intercept only, no covariates; Model 2 adjusted for education, age, sex, country of birth, marital status, self-rated health, chronic disease and physical functional limitation.
3. Wald test that between-area variance for the null model is non zero (i.e. δ_u^2 =0) p<.001 for major cities and inner regional, <.01 for outer regional/remote. See Appendix G for full details of model diagnostics.

Supplementary table 6. Model 2: fixed effects for quality-of-care outcomes by region

	Cities OR (95%CI)	Inner regional OR (95%CI)	Outer regional/ remote OR (95%CI)
**Long consults**			
University (ref.)	1	1	1
Apprentice/diploma	1.00 (0.97–1.04)	0.95 (0.91–0.99)	0.94 (0.90–0.99)
School certificate	0.96 (0.92–0.99)	0.90 (0.86–0.93)	0.90 (0.85–0.94)
No school certificate	0.96 (0.91–1.01)	0.90 (0.86–0.95)	0.92 (0.87–0.98)
**Continuity of care**			
University (ref.)	1	1	1
Apprentice/diploma	1.07 (1.03-1.11)	1.07 (1.02-1.11)	1.06 (1.01-1.11)
School certificate	1.11 (1.07-1.15)	1.18 (1.14-1.23)	1.18 (1.12-1.24)
No school certificate	1.14 (1.07-1.20)	1.24 (1.18-1.31)	1.15 (1.08-1.22)
**Care planning**			
University (ref.)	1	1	1
Apprentice/diploma	1.21 (1.14-1.29)	1.27 (1.19-1.35)	1.25 (1.15-1.36)
School certificate	1.32 (1.24-1.40)	1.40 (1.31-1.49)	1.43 (1.32-1.55)
No school certificate	1.53 (1.42-1.64)	1.64 (1.52-1.76)	1.61 (1.47-1.77)

Notes:

1. GP, general practitioner; OR, odds ratio; CI, confidence interval.
2. Adjusted for sociodemographic (educational attainment, age, sex, country of birth, marital status) and need (self-rated health status, number of chronic disease, physical functioning) variables.
3. Wald joint test of significance for education p<.001 for all outcomes and regions, except long consults in major cities (p<.05).

Supplementary table 7. Model 2: fixed effects for specialist use by region

Model 2, specialist use	Cities OR (95%CI)	Inner regional OR (95%CI)	Outer regional/ remote OR (95%CI)
University (ref.)	1	1	1
Apprentice/diploma	0.94(0.91–0.98)	0.92(0.89–0.96)	0.95(0.91–1.00)
School certificate	0.90(0.86–0.93)	0.91(0.88–0.95)	0.95(0.91–1.00)
No school certificate	0.86(0.81–0.90)	0.85(0.81–0.90)	0.95(0.90–1.01)
p-value for SEP term	<.001	<.001	ns

Notes:

1. OR, odds ratio; CI, confidence interval; SEP, socioeconomic position; ns, not statistically significant.
2. Adjusted for sociodemographic (educational attainment, age, sex, country of birth, marital status) and need (self-rated health status, number of chronic disease, physical functioning) variables.
3. Wald joint test of significance for education cities and inner regional <.001, outer regional not significant.
