# Supplementary material for: Does use of GP and specialist services vary across areas and according to individual socioeconomic position? A multilevel analysis using linked data in Australia
Source: BMJ Open. 2024 Jan 6;14(1):e074624. doi: 10.1136/bmjopen-2023-074624 (PMC10773367; doi:10.1136/bmjopen-2023-074624)
Supplement: Author's manuscript [file bmjopen-2023-074624.draft_revisions.pdf]

BMJ Open is committed to open peer review. As part of this commitment we make the peer review history of every article we publish publicly available.

When an article is published we post the peer reviewers' comments and the authors' responses online. We also post the versions of the paper that were used during peer review. These are the versions that the peer review comments apply to.

The versions of the paper that follow are the versions that were submitted during the peer review process. They are not the versions of record or the final published versions. They should not be cited or distributed as the published version of this manuscript.

BMJ Open is an open access journal and the full, final, typeset and author-corrected version of record of the manuscript is available on our site with no access controls, subscription charges or pay-per-view fees (http://bmjopen.bmj.com).

If you have any questions on BMJ Open's open peer review process please email info.bmjopen@bmj.com

BMJ Open

Area-level and individual-socioeconomic variation in use of GP and specialist services. A multilevel analysis using linked data.

Journal:	*BMJ Open*
Manuscript ID	bmjopen-2023-074624
Article Type:	Original research
Date Submitted by the Author:	12-Apr-2023
Complete List of Authors:	Butler, Danielle; Australian National University<br>Larkins, Sarah; James Cook University Division of Tropical Health and Medicine<br>Jorm, Louisa; University of New South Wales<br>Korda, Rosemary; Australian National University
Keywords:	Primary Health Care, Health Equity, Health policy < HEALTH SERVICES ADMINISTRATION & MANAGEMENT, EPIDEMIOLOGIC STUDIES

SCHOLARONE™
Manuscripts

I, the Submitting Author has the right to grant and does grant on behalf of all authors of the Work (as defined in the below author licence), an exclusive licence and/or a non-exclusive licence for contributions from authors who are: i) UK Crown employees; ii) where BMJ has agreed a CC-BY licence shall apply, and/or iii) in accordance with the terms applicable for US Federal Government officers or employees acting as part of their official duties; on a worldwide, perpetual, irrevocable, royalty-free basis to BMJ Publishing Group Ltd ("BMJ") its licensees and where the relevant Journal is co-owned by BMJ to the co-owners of the Journal, to publish the Work in this journal and any other BMJ products and to exploit all rights, as set out in our licence.

The Submitting Author accepts and understands that any supply made under these terms is made by BMJ to the Submitting Author unless you are acting as an employee on behalf of your employer or a postgraduate student of an affiliated institution which is paying any applicable article publishing charge ("APC") for Open Access articles. Where the Submitting Author wishes to make the Work available on an Open Access basis (and intends to pay the relevant APC), the terms of reuse of such Open Access shall be governed by a Creative Commons licence – details of these licences and which Creative Commons licence will apply to this Work are set out in our licence referred to above.

Other than as permitted in any relevant BMJ Author's Self Archiving Policies, I confirm this Work has not been accepted for publication elsewhere, is not being considered for publication elsewhere and does not duplicate material already published. I confirm all authors consent to publication of this Work and authorise the granting of this licence.

For peer review only

Area-level and individual-socioeconomic variation in use of GP and specialist services. A multilevel analysis using linked data.

Author list

*Danielle C Butler[1], MBBS, MPH, FRACGP, PhD; ORID: 0000-0003-4870-4544

Sarah Larkins[2], MBBS, MPH&TM, FRACGP, PhD; ORCID: 0000-0002-7561-3202

Louisa R Jorm[3], BVSc, MSc. PhD, FAHMS, ORCID: 0000-0003-0390-661X

Rosemary J Korda[1], MAppSc, GradDipPopHlth, PhD, ORID: 0000-0002-9390-2171

Affiliations

1. National Centre for Epidemiology and Population Health, Australian National University
2. College of Medicine and Dentistry, James Cook University
3. Centre for Big Data Research in Health, University of New South Wales

***Corresponding author details**

Email: Danielle.Butler@anu.edu.au

Postal address: Building 62, Mills Rd, The Australian National University, Acton, ACT, Australia, 2601

Word count: 3327

Number of tables: 1

Number of Figures: 2

For peer review only - http://bmjopen.bmj.com/site/about/guidelines.xhtml

Abstract

Objective: Timely access to primary care and supporting specialist care relative to need is essential for health equity. However, use of services can vary according to an individuals' socioeconomic circumstances or where they live. This study aimed to quantify individual socioeconomic variation in GP and specialist use in New South Wales (NSW), accounting for area-level variation in use.

Design: Multilevel logistic regression was used to estimate median odds ratios (MORs) to quantify small-area variation in need-adjusted GP use and quality-of-care and specialist use, and odds ratios (ORs) to quantify associations with individual socioeconomic position (SEP), separately by remoteness.

Setting: Baseline data (2006–2009) from the 45 and Up Study, NSW, Australia, linked to Medicare Benefits Schedule (MBS) and death data (to December 2012).

Participants: 267, 112 adults aged 45 years and older.

Results: GP (MOR=1.32-1.35) and specialist use (1.16-1.18) varied between areas, accounting for individual characteristics. For a given level of need and accounting for area-variation, low-SEP individuals were more likely to be frequent users of GP services (no school certificate vs university, OR=1.63-1.91, depending on remoteness category) and have continuity of care (OR=1.14-1.24), but were less likely to see a specialist (OR=0.85-0.95).

Conclusion: GP and specialist use varied across small-areas in NSW, independent of individual characteristics. Specialist but not GP care was inequitable. Failure to address inequitable specialist use may undermine equity gains within the PC system. Policies should also focus on local variation.

Key words: primary care, equity, variation in care, socioeconomic inequalities, multilevel analysis

For peer review only - http://bmjopen.bmj.com/site/about/guidelines.xhtml

Strengths and limitations

- Our study used a multilevel analytical design which allowed quantification of area- and individual-level variation in primary care and supporting specialist care; quantifying these differing sources of variation is required for ensuring an equitable healthcare system.
- The large sample linked to health utilisation data, allowed quantification of observed use (rather than self-report) after accounting for a range of factors.
- The study sample was limited to those aged 45 year and older from Australia's most populous state and patterns of association may differ for other age groups or in other settings.

For peer review only

For peer review only - http://bmjopen.bmj.com/site/about/guidelines.xhtml

Introduction

Adequate and timely access to primary care relative to need is a specified goal of high performing health systems (1, 2, 3). This is integral to improving average levels of population health, as well as health equity (4). Further, an effective primary care system requires ready access to supporting specialist care. Yet often individuals' socioeconomic circumstances or where they live, as much as their need for care, determine their use of services (5, 6, 7, 8, 9); that is, access to care is inequitable. Examining and quantifying these differing sources of variation in care is essential for directing policy responses for achieving an equitable healthcare system.

There is evidence internationally (10, 11, 12), and to a lesser extent within Australia (7, 8, 9), of socioeconomic variation in use of GP and specialist services. Across most jurisdictions, people who are of low socioeconomic position (SEP) use equal or more GP services for a given level of need relative to their high-SEP counterparts (7, 8, 9, 12). On the other hand, individuals of high-SEP are more likely to see a specialist than those of low-SEP (9, 10, 12). Use of primary care and specialist services also varies geographically. Studies in Australia using aggregated area-level data consistently find increased use of GP and specialist services in major cities compared with more remote areas (5, 8, 13). To date, no Australian studies have examined individual socioeconomic variation in use of primary and specialist services while accounting for area-variation in use of services, or quantified the extent of variation at the area-level, beyond that explained by the characteristics of individuals living in those areas.

The aim of this study was to use large-scale linked data and multi-level analysis (14, 15) to examine the extent to which GP and specialist service use varied at the area-level, having accounted for the characteristics of people who lived in those areas. Further, we quantified variation in use of services according to individual SEP, having accounted for variation in use across areas. In this way, sources of variation in use of GP and specialist services are clarified and indicate directions for reducing unwarranted variation in care.

For peer review only - http://bmjopen.bmj.com/site/about/guidelines.xhtml

Methods

Study population and setting

The Sax Institute's 45 and Up Study is a large prospective cohort study involving 267,153 people aged 45 years and older residing in New South Wales, the most populous state in Australia (16). Participants were randomly sampled from the Services Australia (formerly the Australian Government Department of Human Services) Medicare enrolment database, with over-sampling by a factor of two of individuals aged 80 years and over and people resident in rural areas. Participants enrolled in the study by completing a baseline questionnaire, distributed between 2006 and 2009, and providing consent for 5-yearly questionnaires and linkage to routinely collected health data. Approximately 11% of the total NSW population aged 45 years and older was included in the study, with a response rate of around 18% (17). The study design and details of the questionnaire are reported elsewhere (17).

Data

Sociodemographic and health variables were derived from the self-reported baseline questionnaire. Data from the questionnaire were linked to Medicare Benefits Schedule (MBS) claims data (1 January 2003 – 14 December 2012) provided by Services Australia, and data from the NSW Registry of Births, Deaths and Marriages (RBDM) and the National Death Index (NDI). The MBS claims database includes all claims for subsidised medical and diagnostic services provided by registered medical and other practitioners through the MBS. For each claim for service processed, the MBS data include a range of information, including the date of the service and the item number for the service. Linkage of baseline data from 45 and Up Study participants to MBS data was performed at the Sax Institute through deterministic linkage, using an encrypted version of the Medicare number provided directly by Services Australia.

Probabilistic linkage to NSW RBDM was performed by the Centre for Health Record Linkage (CHeReL) data. Quality assurance data on the CHeReL data linkage show false positive and negative rates of <0.5% and <0.1%, respectively (18).

For peer review only

For peer review only - http://bmjopen.bmj.com/site/about/guidelines.xhtml

Variables

For use of GP services, the main outcome was above-average GP use (no/yes) as a measure of frequent use, defined as eight or more services in the year following completion of the baseline survey, which is broadly consistent with definitions reported in the literature (9, 19). We also examined secondary outcomes relating to types and qualities of GP services that indicate high-quality primary care (1, 2, 3), and that the general population would be eligible to receive. This included: i) any MBS service for a long or prolonged consultation (no/yes) in the follow up period (known to be associated with more problems managed and better outcomes, (20, 21)); ii) continuity of GP care measured by the usual provider continuity index (UPI) (22), calculated as the proportion of GP MBS services with the most frequent provider of total GP MBS services and defined as a UPI of 70% or more. As per standard definitions, the UPI was calculated over a 2-year period and calculated only for those participants who used at least four services in that time; and iii) care planning (no/yes) defined as at least one MBS service for a chronic disease and complex care planning item (including a GP management plan, team care arrangement or review item) in the follow - up period. These items relate to specific MBS funded services that can be claimed for care planning relating to chronic and complex care needs and to enable multidisciplinary coordination of care.

Specialist use was defined as any out-of-hospital MBS specialist service in the follow up period (no/yes). See additional file 1 for full list of MBS items codes included in the outcome measures.

Individual-level characteristics were derived from the 45 and Up baseline questionnaire. Our main exposure variable, SEP, was measured as the highest educational level attained (no school certificate, school certificate, apprenticeship or diploma, and university degree).

To determine need-adjusted use, healthcare need (9, 23) variables included were: self-reported health (excellent, very good, good, fair and poor); physical functioning (no limitation, minor limitation, moderate limitation, severe limitation and a missing category); and number of chronic conditions for the following self-reported conditions – cancer, asthma, hayfever, heart disease, heart attack, angina,

For peer review only

For peer review only - http://bmjopen.bmj.com/site/about/guidelines.xhtml

stroke, diabetes, hypertension, hypercholesterolaemia, arthritis, osteoporosis, anxiety, depression and Parkinson's disease (none, 1-2 chronic conditions, 3 or more chronic conditions).

To account for confounders in the relationship between SEP or healthcare need and use of health services we also included: age (10 age categories from 45 years through to 85 years and over), sex (male/female); country of birth (Australia/NZ, Europe/Nth America, Asia, Africa/Mid East, and other); and marital status (married/defacto or not married/not defacto).

Using Australian Bureau of Statistics (ABS) concordance files, each participant was assigned to a Statistical Area Level 3 (SA3) geography. These areas have populations of between 30,000 and 130,000 persons and are considered representative of communities sharing similar characteristics in terms of services available (additional file 1).

Analysis

Participants were followed for one year after study entry (most had completed entry by 2008) and were included if they had a least one Medicare record, were alive at the end of the follow up period and had a geographical identifier coded to NSW.

Frequencies and proportions were calculated for the sample according to participant characteristics, for the total sample, by education and by outcomes. A series of two-level random intercept multilevel logistic regression models (participants nested within SA3 of residence) were fitted for each outcome. Two model specifications were used: 1) random intercept with no explanatory variables to determine if outcomes varied at the area-level; and 2) Model 1 adjusted for individual education, healthcare need and confounders to determine need-adjusted individual-level socioeconomic variation in outcomes (having accounted for area-level variation).

Area-level variation in each outcome was estimated from the variance term (V_A) by calculating the ICC by the linear threshold model method (ICC=$V_A/(V_A+3.29)$ and the median odds ratio (MOR=exp $(0.954\sqrt{V_A})$ (14). The proportional change in variance (PCV=($V_A - V_B/V_A$) x 100) (14) was used to estimate the proportion of overall variation in outcome explained by addition of explanatory variables

For peer review only - http://bmjopen.bmj.com/site/about/guidelines.xhtml

to the model. Second-order penalised quasi-likelihood (PQL) estimation was used as per Rasbash and colleagues (15). Monte Carlo Markov Chain (MCMC) estimation was used to assess model fit statistics and residuals plotted to test model assumptions held.

As health service use in Australia varies according to remoteness, analyses were stratified by categories of remoteness (major cities, inner regional, outer regional/remote) based on the 2006 Access and Remoteness Index of Australia (+) (24) and according to the Australian Statistical Geography Standard Classification of remoteness (ASGC-RA).

Analyses were undertaken using Stata (College Station, Texas, StataCorp; Version 14.1) in the Secure Unified Research Environment, a secure remote-access computer facility for analysis of linked data. Multilevel analysis were performed using the runmlwin add-on (25), using Stata's post estimation procedures.

Sensitivity analyses were also repeated using alternative measures of frequency of GP use (low versus medium and medium versus high) and including those who died in the follow up period.

Ethics approval for this project was obtained from the NSW Population and Health Services Research Ethics Committee (HREC/13/CIPHS/8), the University of Western Sydney Ethics Committee (H9835) and the Australian National University Human Research Ethics Committee (2011/703). Ethics approval for the 45 and Up Study was granted by the University of New South Wales Human Research Ethics Committee. The 45 and Up Study participants consented to data linkage at baseline. Linkage of the MBS data is performed under approvals from the ethics committees of Services Australia and the Australian Government Department of Health.

Patient and public involvement

No patients or members of the public were involved in the design or conduct of the study.

Results

Sample Characteristics

For peer review only - http://bmjopen.bmj.com/site/about/guidelines.xhtml

For peer review only

After excluding those who had an invalid death date or died in the follow up period (n=320), did not have an MBS service (n=1583), or were unable to be assigned to an SA3 (n=151) the final sample for inclusion was 263,083. Of these, 11.7% had no school certificate, 31.8% completed a school certificate, 31.8% had completed an apprenticeship or diploma and 23% had completed a tertiary level qualification. The mean age of the population was 62.7 years (SD 11.2), 46% were male, over 80% rated their health as good, very good or excellent and 73% had at least one chronic condition (Table 1).

[Table 1 here]

Area-level variation

Use of GP services varied according to where a person resided–for all regions–having accounted for the characteristics of individuals living in those areas (Figure 1, MOR major cities 1.34, inner regional 1.32, outer regional/remote 1.35). This means that an individual who lived in area with a higher rate of above-average GP use had a (median) 32-34% greater probability of having above-average GP use than an individual with identical characteristics who lived in an area with a lower rate of above-average GP use. Area-level variation in specialist use across all regions was also evident after accounting for the characteristics of individuals (MOR 1.16–1.17; additional file 1).

[Figure 1 here]

Individual level socioeconomic variation

For a given level of need, people of low education used more GP services on average compared to those with higher levels of education, having accounted for area variation in use (no school certificate vs university educated; major cities OR 1.91, 95%CI [1.81, 2.03], inner regional 1.63 [1.54, 1.73], outer regional remote 1.72 [1.60, 1.84], Figure 2). For secondary outcomes examining quality of GP care, people of low education were also more likely to have care planning (e.g. no school certificate vs university educated in major cities 1.53[1.42, 1.14]) and continuity of care (e.g. in major cities 1.14[1.07, 1.20]) compared to their high education counterparts, but less likely to have a long

For peer review only - http://bmjopen.bmj.com/site/about/guidelines.xhtml

consultation (e.g. inner regional 0.90[0.87, 0.95]), accounting for area-variation in these outcomes (additional file 1). Patterns of association were found whether in major cities or more remote locations.

[Figure 2 here]

On the other hand, people of low education (for a given level of need) were less likely have a specialist service compared to their higher-education counterparts, accounting for area-variation (no school certificate vs university educated; major cities 0.86 [0.81, 0.90], inner regional 0.85 [0.81, 0.90], Figure 2, additional file 1).

Sensitivity analyses did not differ materially from the main findings.

Discussion

This study has shown, that where people live (at the local area-level) matters for the GP and specialist services they receive, independent of their personal characteristics. This was the case across all remoteness categories - major cities, regional and more remote areas - in New South Wales. Further, having accounted for where people live, use of GP services and quality of care was equitable, in that disadvantaged people were more likely to use more services on average, and to have continuity of care and care planning. However, the finding that advantaged people were more likely to see a specialist or have a long consultation suggests a potential source of inequity.

This is the first study in Australia and one of few internationally to quantify area-level variation in GP and specialist use, independent of the characteristics of people who lived in these areas. The amount of variation between areas quantified in this study is comparable to that previously reported when examining other healthcare outcomes in Australia (e.g. hospitalisations (26)), and internationally (10). More use of GP services and care planning and greater continuity of care among people of lower SEP has been previously shown in Australia (7, 8, 9, 27, 28) and internationally (29, 30). This study confirms that this holds having accounted for where people live. People of lower SEP are more likely to have

For peer review only - http://bmjopen.bmj.com/site/about/guidelines.xhtml

multiple and complex health and psychosocial care needs (31) than their advantaged counterparts; continuity and care planning are essential for enabling these needs to be met. International data from countries without gate-keeping mechanisms in place have found inequity of specialist use (10), independent of where people live. Our study demonstrates this was also found within a setting with gate-keeping policies in place.

We found that individual use of GP and specialist services varied across small-areas, for all remoteness categories, beyond what could be explained by the characteristics of people living in those areas. This suggests that there are aspects within peoples' local context that systematically shape the care of all who live in that area. The specific reasons are unknown but may relate to how services are organised and delivered (including availability of providers) within an area or structural policies determining the geographical distribution of services and providers. International multilevel studies in countries with (32) and without (10) a gate-keeping mechanism have shown that availability of GPs and specialists within an area was associated with specialist use. This has not been investigated for GP service use or quality of care. Importantly, how services are organised can be changed (through policy and practice) and doing so may contribute to reducing the unwarranted variation across areas.

There are likely multiple reasons why socioeconomically disadvantaged people use less specialist services for a given level of need. Unlike GP care, in Australia there are no bulk-billing incentives for specialists. Out-of-pocket costs for specialist services doubled in the decade prior to the study period (33) and have continued to rise since. Further, private health insurance has been shown to contribute to pro-high income use of specialist services (9); yet government funded rebates for private health insurance have remained in place. Other possible reasons include: differences in propensity to seek care due to differences in health literacy, attitudes and beliefs; or, due to negatively biased behaviours from providers, disadvantaged people are less likely to seek specialist care (34). However, if this was the case a similar finding would be expected with use of GP services. Further, studies examining propensity to seek care (35) or rates of completion of specialist referrals(36) have not found differences between socioeconomic groups.

For peer review only - http://bmjopen.bmj.com/site/about/guidelines.xhtml

Alternatively, these differences may be due to provider preferences and bias. International evidence also suggests providers offer fewer services to those of low SEP (34) and are more likely to refer higher SEP individuals to a specialist (37). Irrespective of the reasons, differences in use does not reflect need for care and hence is inequitable and unjust.

The reason why people of high education were more likely to have a long consultation is unknown. Possibly, low educated people are more likely to be bulk-billed, and given current financing arrangements in Australia, the benefit per minute falls with longer consultations. These findings may also reflect differences in health literacy. More highly educated people may be more likely to anticipate and expect a range a health issues to be addressed in a single episode and request a consultation length to that effect, or actively seek out practitioners with characteristics associated with longer consultations(38).

A strength of our study is the multilevel analytical design, which allowed modelling of nested levels of data and quantification of area- and individual-level variation. Further, the large sample linked to MBS service use, allowed quantification of observed use (rather than self-report) after accounting for a range of factors. While MBS data will capture nearly all GP services, there are some settings where services provided do not attract an MBS claim. For example, publicly funded community health centres and some GP services provided in emergency departments in rural and remote areas. In addition, a substantial proportion of specialist services in Australia are provided in publicly funded hospital-based outpatient clinics, which generally do not attract an MBS rebate. Low-SEP people are more likely to use these community and hospital-based services (9) and exclusion of these services may bias estimates for SEP gradients to be pro-high SEP. However, previous studies found this did not alter estimates of socioeconomic variation in GP and ambulatory specialist care(9). The 45 and Up study is not representative of the NSW population (39), and while representativeness is not necessary for internal validity (i.e. relative effect estimates) (39), patterns of association may differ for other age groups or in other settings.

For peer review only

For peer review only - http://bmjopen.bmj.com/site/about/guidelines.xhtml

Implications for policy and practice.

An effective PC system requires ready and reliable access to secondary level care. This has not been equitably achieved in Australia–despite the presence of universal health insurance–undermining the equity that exists in the PC system. National structural policies, such as minimising out-of-pocket costs for example through bulk-billing incentives, would go some way to redressing inequitable use of specialist services. Given that private health insurance contributes to pro-high SEP use of specialist services, offsetting government rebates in favour of lower income or disadvantaged individuals could also contribute to reducing this inequity. It could be argued that the inequity in community-based specialist services is balanced by a pro-low SEP preference for specialist outpatient services through the public hospital sector. However, waiting times for less urgent and more discretionary health needs (and in some instances for more urgent health needs) in the public sector are understood to exceed that in the private sector (40), although actual wait times are not published. This increases the impact of illness on recovery and quality of life, affecting those who are disadvantaged to a greater extent. As such, addressing inequalities in access to specialist care is even more pressing.

The unwarranted variation in both GP and specialist use at the area-level suggests that additional policy approaches are needed that are directed to local contexts, in addition to individuals. For example, it may be that availability of providers (both GPs and specialists) may need to be addressed, as international studies have shown this explains some of the area-level variation in care. Similarly, there may be other aspects of how services are organised and delivered at the local area-level that may determine peoples' use of services. The specific drivers and hence policy solutions to addressing the unwarranted area-level variation requires further exploration.

Conclusion

It is reassuring that, for a given level of need, GP service use and important aspects of quality of care (such as care planning, continuity of care) favours those who are disadvantaged; further, this is the case regardless of where people live. However, the ongoing pro-high SEP use of specialist service

For peer review only - http://bmjopen.bmj.com/site/about/guidelines.xhtml

threatens to undermine this and requires urgent attention. Equity measures to improve affordability are an important avenue to address this. However, both GP and specialist care varies not only between major cities and more remote locations, but also within at the small area-level. This is unwarranted and highlights an important opportunity to improve equity in the Australian healthcare system.

For peer review only

For peer review only - http://bmjopen.bmj.com/site/about/guidelines.xhtml

Table 1. Sample characteristics: individual-level variables by educational attainment (%) and for total sample

	Educational attainment					
**Variable**	**No school certificate**	**School certificate**	**Apprentice/ diploma**	**University**	**Missing**	**Row category total %(n)**
Education						
Total %(n)	11.7(31,126)	31.8(84,302)	31.8(84,294)	23(60,933)	1.7(4,428)	100(265,083)
Sex						
Male	42.1	36.1	55.2	50.0	48.6	46(122,893)
Female	57.3	63.9	44.8	50.0	51.5	53.6(142,190)
Age						
45–54	16.5	24.4	31.6	40.1	14.4	29.2(77,397)
55–64	27.4	32.9	31.9	34.8	22.6	32.2(85,342)
65–74	28.9	23.7	21.5	15.7	25.4	21.8(57,734)
75–84	21.8	15.3	12.7	8.0	28.8	13.8(36,516)
85 plus	5.4	3.8	2.3	1.5	8.8	3.1(8,082)
Country of birth						
Australia/NZ	75.9	80.8	76.9	72.5	64.8	76.8(203,629)
Europe/ N. America	18.6	13.4	17.7	16.8	20.9	16.3(43,154)
Asia	2.3	2.6	2.3	6.5	4.0	3.4(9,031)
Africa/Mid. East	1.1	1.5	1.3	2.7	1.7	1.7(4,397)
Other	0.5	0.8	0.9	0.8	0.9	0.08(2,145)
Marital status						
Not married/ not de facto	32.6	26.2	22.3	21.0	33.5	24.7(65,288)
Married/de facto	66.8	73.3	77.1	78.5	64.3	74.7(198,185)
Self-rated health						
Excellent	7.4	12.2	14.1	22.4	10.1	14.6(38,575)
Very good	25.6	35.0	36.8	40.8	24.6	35.6(94,481)
Good	36.3	34.5	33.8	26.5	32.1	32.6(86,451)
Fair	20.5	12.3	10.7	6.9	17.5	11.6(30,644)
Poor	4.8	2.2	1.8	1.0	4.0	2.1(5,575)

For peer review only

For peer review only - http://bmjopen.bmj.com/site/about/guidelines.xhtml

Variable	Educational attainment					
	No school certificate	School certificate	Apprentice/ diploma	University	Missing	Row category total %(n)
Chronic conditions						
none	20.7	25.4	27.3	31.2	25.2	26.8(70,991)
1–2	49.9	52.0	52.6	53.0	50.1	52.1(198,116)
3 or more	29.4	22.6	20.2	15.8	24.7	21.1(55,976)
Physical functioning						
No limitation	18.9	26.5	30.0	39.4	19.2	29.5(78,323)
Minor limitation	15.4	23.2	26.9	30.2	14.5	24.9(66,072)
Moderate limitation	21.4	22.5	21.6	17.3	16.7	20.8(55,097)
Severe limitation	21.8	12.9	10.3	5.5	16.5	11.5(30,367)
GP use						
Below average (%)	46.5	59.0	64.4	74.3	47.8	62.6(165,803)
Above average (%)	53.6	41.1	35.6	25.7	52.2	37.5(99,280)
Continuity of care						
<70%	41.1	44.8	46.5	50.4	41.6	46.1(105,433)
≥70%	58.7	55.0	53.4	49.6	58.1	53.7(128,055)
Care planning						
No	53.5	54.8	55.3	56.3	51.6	55.1(146,046)
Yes	22.4	15.9	13.5	8.5	20.1	14.3(37,815)
Long consult						
No	56.5	58.7	60.1	59.8	56.2	59.1(156,652)
Yes	43.5	41.3	39.9	40.2	43.8	40.9(108,428)
Any specialist use						
No (%)	40.5	44.3	46.5	47.9	40.5	45.3(120,063)
Yes (%)	59.5	55.7	53.6	52.1	59.5	54.7(145,019)

Notes: N, number; %, percentage; Columns for each variable category for each educational attainment categories sum to 100%. Values in last column gives break down by category for each individual variables for the total sample, not stratified by educational attainment. For each variable, total (n) sums to 265,083 and percent sums to 100% including missings;Chi-squared test for trend with education p<.001 all variables. Missing: age<1%, country of birth 1%, marital status 0.6%, self-rated health 3.5%, physical functioning 13.3%, continuity of care 0.1%, care planning 30.6%. 3. Missing for care planning includes those excluded as ineligible (i.e. do not have a chronic disease or long-term condition).

For peer review only - http://bmjopen.bmj.com/site/about/guidelines.xhtml

Figure titles and legends

Figure 1. Difference between mean for each area (SA3) and the mean across all areas in log odds of above-average use (95% CI) for each area, by remoteness

Notes: Adjusted for education, age, sex, country of birth, marital status, self-rated health, chronic disease and physical functional limitation; Mean log odds of above-average use across areas for that remoteness category set 0 and given by the horizontal red line; Each dot represents the mean for each SA3 of the difference in log odds of above-average use for each person in that SA3 from the mean log odds of above-average use for all areas (i.e. the mean of the residuals by SA3). Bars are the 95% confidence intervals around the mean for each SA3. SA3 values that lie above and below the red with CIs that do not cross the red line, are significantly different from the mean log odds of above-average use for all SA3s in that remoteness category. A person living in an area above the line has a higher probability of above-average GP use than the overall sample mean, irrespective of their individual characteristics; CI, confidence interval; SA3, statistical area 3.

Figure 2. Odds ratios and 95% confidence intervals for education with above-average use of GP services and any use of specialist services, by remoteness.

Notes: GP, general practitioner; OR, odds ratio; CI, confidence interval; %, percentage; ref., reference. Model fitted with a random intercept (area-level) adjusted for sociodemographic (education, age, sex, country of birth, marital status) and need (self-rated health status, number of chronic disease, physical functioning) variables. GP use, Wald joint test of significance for education p<.001 for all remoteness categories. Any specialist use Wald joint test of significance for education cities and inner regional <.001, outer regional not significant.

Supplementary Information

Additional file 1:

References

1. olde Hartman TC, Bazemore A, Etz R, et al. Developing measures to capture the true value of primary care. BJGP Open. 2021:BJGPO.2020.0152.

2. Veillard J, Cowling K, Bitton A, et al. Better Measurement for Performance Improvement in Low- and Middle-Income Countries: The Primary Health Care Performance Initiative (PHCPI) Experience of Conceptual Framework Development and Indicator Selection. Milbank Q. 2017;95(4):836-83.

3. Kringos DS, Boerma WG, Bourgueil Y, et al. The European primary care monitor: structure, process and outcome indicators. BMC Fam Pract. 2010;11:81.

4. Solar O, Irwin A. A conceptual framework for action on the social determinants of health. Social Determinants of Health discussion paper 2 (policy and practice). Geneva: World Health Organisation; 2010.

For peer review only

For peer review only - http://bmjopen.bmj.com/site/about/guidelines.xhtml

5. Mu C, Hall J. What explains the regional variation in the use of general practitioners in Australia? BMC Health Services Research. 2020;20(1):325.

6. Haggerty J, Levesque J-F, Harris M, et al. Does healthcare inequity reflect variations in peoples' abilities to access healthcare? Results from a multi-jurisdictional interventional study in two high-income countries. Intern. 2020;19(1):167.

7. Ou L, Chen J, Hillman K. Socio-demographic disparities in the utilisation of general practice services for Australian children - Results from a nationally representative longitudinal study. PloS one. 2017;12(4):e0176563.

8. Turrell G, Oldenburg BF, Harris E, et al. Utilisation of general practitioner services by socio-economic disadvantage and geographic remoteness. Aust N Z J Public Health. 2004;28(2):152-8.

9. Korda RJ, Banks E, Clements MS, et al. Is inequity undermining Australia's 'universal' health care system? Socio-economic inequalities in the use of specialist medical and non-medical ambulatory health care. Aust Nz J Publ Heal. 2009;33(5):458-65.

10. Chaix B, Boelle PY, Guilbert P, Chauvin P. Area-level determinants of specialty care utilization in France: a multilevel analysis. Public Health. 2005;119(2):97-104.

11. Beckman A, Anell A. Changes in health care utilisation following a reform involving choice and privatisation in Swedish primary care: a five-year follow-up of GP-visits. BMC Health Serv Res. 2013;13:452.

12. Devaux M, de Looper M. Income related inequalities in health service utilisation in 19 OECD countries, 2008-2009. OECD Publishing, Paris. http://dx.doi.org/10.1787/5k95xd6stnxt-en; 2012.

13. AIHW. Medicare-subsided GP, allied health and specialist care across local areas: 2013-14 to 2018-19 Canberra2020 [7 August 2021]. Available from: https://www.aihw.gov.au/reports/primary-health-care/medicare-subsidised-health-local-areas-2019/contents/introduction.

14. Merlo J, Chaix B, Ohlsson H, et al. A brief conceptual tutorial of multilevel analysis in social epidemiology: using measures of clustering in multilevel logistic regression to investigate contextual phenomena. J Epidemiol Community Health. 2006;60(4):290-7.

For peer review only - http://bmjopen.bmj.com/site/about/guidelines.xhtml

15. Rasbash J, Steel BJ, Browne WJ, et al. A user's guide to MLwin. University of Bristol: Centre for Multilevel Modelling; 2012.

16. ABS. 2071.0 Census of population and housing: Reflecting Australia-stories from the Census, 2016. Canberra: ABS; 2016.

17. Banks E, Redman S, Jorm L, et al. Cohort profile: the 45 and up study. Int J Epidemiol. 2008;37(5):941-7.

18. NSW Centre for Health Record Linkage. 2018 [Available from: http://www.cherel.org.au/.

19. Smits FTM, Mohrs JJ, Beem EE, Bindels PJE, van Weert HC. Defining frequent attendance in general practice. BMC Fam Pract. 2008;9.

20. Wilson A, Childs S. The relationship between consultation length, process and outcomes in general practice: a systematic review. Br J Gen Pract. 2002;52(485):1012-20.

21. Britt HC, Valenti L, Miller GC. Determinants of consultation length in Australian general practice. Med J Aust. 2005;183(2):68-71.

22. Jee SH, Cabana MD. Indices for continuity of care: a systematic review of the literature. Medical care research and review : MCRR. 2006;63(2):158-88.

23. Andersen RM. Revisiting the behavioral model and access to medical care: does it matter? J Health Soc Behav. 1995;36(1):1-10.

24. Australian Bureau of Statistics. Remoteness Structure 2015 [Available from: http://www.abs.gov.au/websitedbs/d3310114.nsf/home/remoteness+structure.

25. Leckie G, Charlton C. runmlwin: A program to run the MLwiN multilevel modeling software from within Stata. Journal of Statistical Software. 2012;52(11):1-40.

26. Falster MO, Jorm LR, Douglas KA, et al. Sociodemographic and health characteristics, rather than primary care supply, are major drivers of geographic variation in preventable hospitalizations in Australia. Med Care. 2015;53(5):436-45.

For peer review only

For peer review only - http://bmjopen.bmj.com/site/about/guidelines.xhtml

27. Douglas KA, Yen LE, Korda RJ, et al. Chronic disease management items in general practice: a population-based study of variation in claims by claimant characteristics. Med J Australia. 2011;195(4):198-202.

28. McRae I, Yen L, Gillespie J, et al. Patient affiliation with GPs in Australia--who is and who is not and does it matter? Health Policy. 2011;103(1):16-23.

29. Kristjansson E, Hogg W, Dahrouge S, et al. Predictors of relational continuity in primary care: patient, provider and practice factors. BMC Fam Pract. 2013;14:72.

30. Jatrana S, Crampton P, Richardson K. Continuity of care with general practitioners in New Zealand: results from SoFIE-Primary Care. The New Zealand medical journal. 2011;124(1329):16-25.

31. Stevenson AD, Phillips CB, Anderson KJ. Resilience among doctors who work in challenging areas: a qualitative study. Br J Gen Pract. 2011;61(588):e404-10.

32. Chan BT, Austin PC. Patient, physician, and community factors affecting referrals to specialists in Ontario, Canada: a population-based, multi-level modelling approach. Med Care. 2003;41(4):500-11.

33. Australian Government Department of Health. Annual medicare statistics financial year 1984-85 to 2015-16 2017 [Available from: http://www.health.gov.au/internet/main/publishing.nsf/content/annual-medicare-statistics.

34. Dovidio JF, Fiske ST. Under the Radar: How Unexamined Biases in Decision-Making Processes in Clinical Interactions Can Contribute to Health Care Disparities. American journal of public health. 2012;102(5):945-52.

35. van Loenen T, van den Berg MJ, Faber MJ, et al. Propensity to seek healthcare in different healthcare systems: analysis of patient data in 34 countries. BMC Health Services Research. 2015;15(1):465.

36. Forrest CB, Shadmi E, Nutting PA, et al. Specialty Referral Completion Among Primary Care Patients: Results From the ASPN Referral Study. Ann Fam Med. 2007;5(4):361-7.

For peer review only

For peer review only - http://bmjopen.bmj.com/site/about/guidelines.xhtml

37. Sørensen TH, Olsen KR, Vedsted P. Association between general practice referral rates and patients; socioeconomic status and access to specialised health care. Health Policy.92(2):180-6.

38. Wilson A, Childs S. The effect of interventions to alter the consultation length of family physicians: a systematic review. Br J Gen Pract. 2006;56(532):876-82.

39. Mealing NM, Banks E, Jorm LR, et al. Investigation of relative risk estimates from studies of the same population with contrasting response rates and designs. BMC Med Res Methodol. 2010;10:26.

40. Public-private time the key to reining in public hospital specialist wait lists [press release]. Univerity of Melbourne2016.

Declarations

Ethics approval and consent to participate

Ethics approval for this project was obtained from the NSW Population and Health Services Research Ethics Committee (HREC/13/CIPHS/8), the University of Western Sydney Ethics Committee (H9835) and the Australian National University Human Research Ethics Committee (2011/703). Ethics approval for the 45 and Up Study was granted by the University of New South Wales Human Research Ethics Committee. The 45 and Up Study participants consented to data linkage at baseline. Linkage of the MBS data is performed under approvals from the ethics committees of Services Australia and the Australian Government Department of Health.

Consent for publication

Not applicable

Availability of data and materials

The data that support the findings of this study are available from the Sax Institute, NSW but restrictions apply to the availability of these data, which were used under license for the current study, and so are not publicly available. Data part of the Sax Institute's 45 and Up Study are available for approved projects to approved researchers (www.saxinstitute.org.au).

For peer review only

For peer review only - http://bmjopen.bmj.com/site/about/guidelines.xhtml

Competing interests

The authors declare that they have no competing interests.

Funding

This research was supported through a grant from the Australian Government through the National Health and Medical Research Council Postgraduate Scholarship (GNT1038903).

Authors' contributions

DB, LJ, SL, RK conceived and designed the analysis. DB completed data analysis and drafted the manuscript. All authors revised the work for intellectual content and approved the final version of the manuscript.

Acknowledgements

This research was completed using data collected through the 45 and Up Study (www.saxinstitute.org.au). The 45 and Up Study is managed by the Sax Institute in collaboration with major partner Cancer Council NSW; and partners: the Heart Foundation; NSW Ministry of Health; NSW Department of Communities and Justice; and Australian Red Cross Lifeblood. We thank the many thousands of people participating in the 45 and Up Study.

For peer review only

For peer review only - http://bmjopen.bmj.com/site/about/guidelines.xhtml

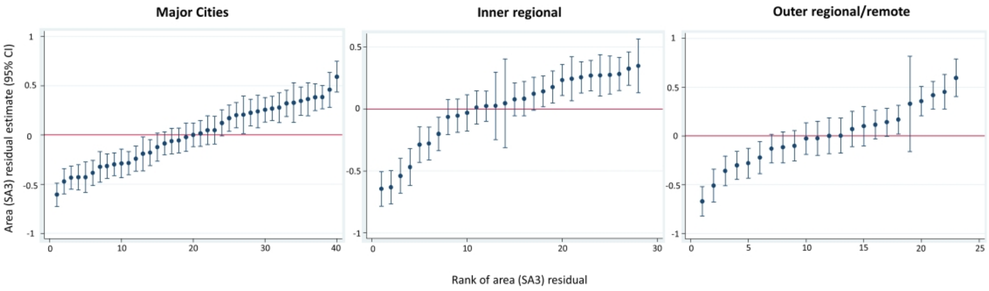

Figure 1. Difference between mean for each area (SA3) and the mean across all areas in log odds of above-average use (95% CI) for each area, by remoteness

Notes: Adjusted for education, age, sex, country of birth, marital status, self-rated health, chronic disease and physical functional limitation; Mean log odds of above-average use across areas for that remoteness category set 0 and given by the horizontal red line; Each dot represents the mean for each SA3 of the difference in log odds of above-average use for each person in that SA3 from the mean log odds of above-average use for all areas (i.e. the mean of the residuals by SA3). Bars are the 95% confidence intervals around the mean for each SA3. SA3 values that lie above and below the red with CIs that do not cross the red line, are significantly different from the mean log odds of above-average use for all SA3s in that remoteness category. A person living in an area above the line has a higher probability of above-average GP use than the overall sample mean, irrespective of their individual characteristics; CI, confidence interval; SA3, statistical area 3.

338x190mm (300 x 300 DPI)

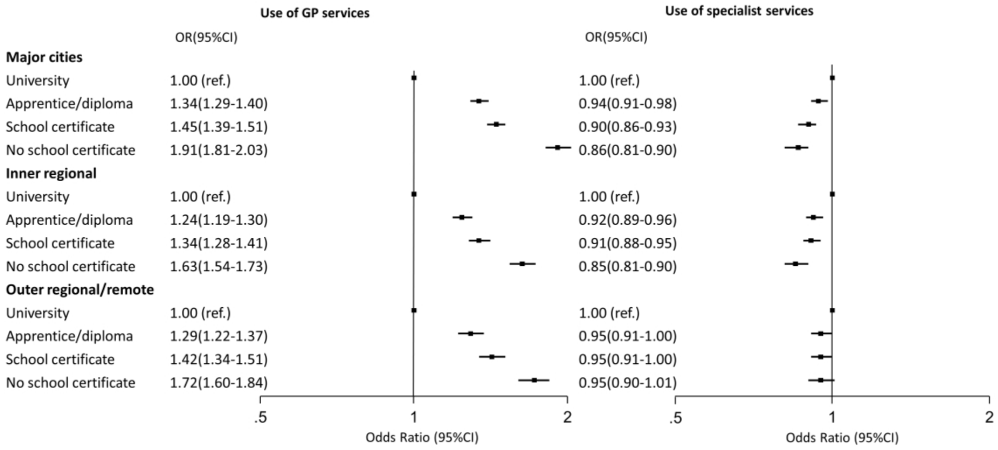

Figure 2. Odds ratios and 95% confidence intervals for education with above-average use of GP services and any use of specialist services, by remoteness.
Notes: GP, general practitioner; OR, odds ratio; CI, confidence interval; %, percentage; ref., reference. Model fitted with a random intercept (area-level) adjusted for sociodemographic (education, age, sex, country of birth, marital status) and need (self-rated health status, number of chronic disease, physical functioning) variables. GP use, Wald joint test of significance for education p<.001 for all remoteness categories. Any specialist use Wald joint test of significance for education cities and inner regional <.001, outer regional not significant.

338x190mm (300 x 300 DPI)

Title: Area-level and individual-socioeconomic variation in use of GP and specialist services. A multilevel analysis using linked data.

Supplementary table 1: Item numbers for GP services

MBS item groups and name of group	Specific item numbers within item group	Notes on item numbers
A1-GP attendances	3, 23, 36, 44<br><br>Remainder A1 attendances=4, 20, 24, 35, 37, 43, 47, 51	Items for services in consulting rooms<br>Items relate to consultations at residential care OR other than residential care/consulting rooms
A2-MPs other than GP	52,53,54,57<br><br>Remainder of A2 attendances=58–60, 65, 92, 93, 95, 96	Items for services in consulting rooms<br>Items relate to consultations at residential care OR other than residential care/consulting rooms
A11-GP	597, 599	Urgent after-hours care
A11-MP other than GP	598, 600	Urgent after-hours care
A14-heath Assessments	701, 703, 705, 707	
A14-health assessments for Aboriginal and Torres Strait Islander peoples	715	
A15-GP management plans	721, 723, 729, 731, 732	Care plans, care plan reviews and team care arrangements
A15-case conference	735, 739, 743, 747, 750, 758	
A17	900 and 903	Medication reviews
A18-GP, cervical screening	2497, 2501, 2503, 2504, 2506, 2507, 2509	Incentive for overdue pap smears (>4 years since last pap)
A18-GP asthma	2546, 2547, 2552, 2553, 2558, 2559	cycle of care
A18-GP diabetes	2517, 2518, 2521, 2522, 2525, 2526	cycle of care
A19-non-referred cervical screening	2598, 2600, 2603, 2606, 2610, 2613, 2616	As per A19 for non-VR MPs
A19-non-referred, asthma	2664, 2666, 2668, 2673, 2675, 2677	As for A18 for non-VR MPs
A19-non-referred, diabetes	2620, 2622, 2624, 2631, 2633, 2635	As for A18 for non-VR MPs
A20-GP mental health care plans	2700, 2701, 2712, 2713, 2715, 2717, 2721, 2723, 2725, 2727	Preparation and review of mental health care plans
A22-after hours (GP attendances)	5000, 5003, 5010, 5020, 5023, 5028, 5040, 5043, 5049, 5060, 5063, 5067	Includes consultation room, residential facility and other. At consultation rooms bolded
A23-after hours (non-referred)	5200, 5203, 5207, 5208, 5220, 5223, 5227, 5228, 5260, 5263, 5265, 5267	As per A22 for non-VR MPs

Notes:

1. GP, general practitioner; MP, medical practitioner; VR vocationally registered.
2. All item numbers were included for frequency of GP use and continuity of care outcome measures. The specific item numbers included for length of consultation were 36, 44, 54, and 57. The specific item numbers for care planning included all those listed under A15 GP management plans.
3. Medical practitioners (MPs) in this case refer to medical doctors who provide non-referred services but are not vocationally registered as a GP (i.e. have not undertaken specialist training in General Practice or other equivalent recognised training program).

For peer review only

Supplementary table 2. Item numbers included for specialist use

MBS item groups and name of group	Specific item numbers within item group	Notes on item numbers
A3-specialist	99, 104, 105, 106, 107, 108, 109, 113	includes home visits; visits at surgery or hospital 99; 113=telehealth
A4-consultant physician	110, 112, 114, 116, 119, 122, 128, 131, 132, 133	112 & 114 telehealth; 122=home visit
A28-geriatric medicine	141, 143,145, 147, 149	145=home visit; 149 telehealth
A8-consultant psychiatry	288, 291, 293, 296, 299, 300, 302, 304, 306, 308, 310, 312, 314, 316, 318, 319, 330, 332, 334, 336, 338, 342, 344, 346, 353, 355, 356, 357, 358, 359, 361, 364, 366, 367, 369, 370	288=telehealth; 289 not included, for autism developmental delay, referred from any practitioner; 299=home visit, hospital consults excluded, interviewing other than patient excluded; 353–370 telepsychiatry
A12-occupational physician	384, 385, 386, 387, 388, 389	
A13-public health	410, 411, 412, 413, 414, 415, 416, 417	Case conferences excluded
A24-pain and palliative medicine	2799, 2801, 2806, 2814, 2820, 2824, 2832, 2840, 3003, 3005, 3010, 3014, 3015, 3018, 3023, 3028	Case conferences excluded
A26-neurosurgery	6004, 6007, 6009, 6011, 6013, 6015, 6016	

1. Note:
2. A3-specialist also includes all attendances by surgeons (at surgery/consulting rooms or hospital). With respect to surgical specialists, only neurosurgery has specific item numbers for attendances.

Supplementary table 3. Between-area variation in odds of above-average GP use.

	Null model	Model 2
**Cities**		
ICC	0.044	0.028
PCV (%)	na	36
MOR	1.45	1.34
**Inner regional**		
ICC	0.023	0.026
PCV (%)	na	-13
MOR	1.30	1.32
**Outer regional/remote**		
ICC	0.022	0.029
PCV (%)	na	-32
MOR	1.29	1.35

Notes:
1. ICC, intra-class coefficient; PCV, proportional change in variance (reported change from null model); MOR, median odds ratio.
2. Null model, random intercept only, no covariates; Model 2 adjusted for education, age, sex, country of birth, marital status, self-rated health, chronic disease and physical functional limitation.
3. Wald test that between-area variance for the null model is non zero (i.e. δ_u^2 =0) p<.001. See Appendix G for full details of model diagnostics.

Supplementary table 4. Between-area variation in odds of quality of GP care outcomes

	Long consults		Continuity of care		Care planning	
	**Null model**	**Model 2**	**Null model**	**Model 2**	**Null model**	**Model 2**
**Cities**						
ICC	0.009	0.013	0.007	0.007	0.049	0.039
PCV (%)	na	-44	na	1.5	na	22
MOR	1.18	1.21	1.16	1.16	1.48	1.41
**Inner regional**						
ICC	0.027	0.031	0.017	0.019	0.049	0.048
PCV (%)	na	-16	na	-8	na	4
MOR	1.33	1.36	1.26	1.27	1.48	1.47
**Outer regional**						
ICC	0.033	0.041	0.037	0.037	0.043	0.044
PCV (%)	na	-24	na	-0.4	na	-2.1
MOR	1.37	1.43	1.40	1.40	1.44	1.45

Notes:

1. ICC: intra-class coefficient; PCV: proportional change in variance (reported change from null model), MOR median odds ratio; na, not applicable.
2. Null model, random intercept only, no covariates;Model 2 adjusted for education, age, sex, country of birth, marital status, self-rated health, chronic disease and physical functional limitation.
3. Wald test that between-area variance for the null model is non zero (i.e. δ_u^2 =0) p<.001 for all outcomes. See Appendix G for full details of model diagnostics

Supplementary table 5. Between-area variation in the odds of specialist use

	**Null model**	**Model 2**
**Major cities**		
ICC	0.007	0.007
PCV (%)	na	-8
MOR	1.15	1.16
**Inner regional**		
ICC	0.008	0.009
PCV (%)	na	-19
MOR	1.17	1.18
**Outer regional**		
ICC	0.008	0.008
PCV (%)	na	-8
MOR	1.16	1.17

Notes:

1. ICC: intra-class coefficient; PCV: proportional change in variance. Reported change from null model; MOR, median odds ratio; na, not applicable.
2. Null model, random intercept only, no covariates; Model 2 adjusted for education, age, sex, country of birth, marital status, self-rated health, chronic disease and physical functional limitation.
3. Wald test that between-area variance for the null model is non zero (i.e. δ_u^2 =0) p<.001 for major cities and inner regional, <.01 for outer regional/remote. See Appendix G for full details of model diagnostics.

For peer review only

Supplementary table 6. Model 2: fixed effects for quality-of-care outcomes by region

	Cities<br>OR (95%CI)	Inner regional<br>OR (95%CI)	Outer regional/ remote<br>OR (95%CI)
**Long consults**			
University (ref.)	1	1	1
Apprentice/diploma	1.00 (0.97–1.04)	0.95 (0.91–0.99)	0.94 (0.90–0.99)
School certificate	0.96 (0.92–0.99)	0.90 (0.86–0.93)	0.90 (0.85–0.94)
No school certificate	0.96 (0.91–1.01)	0.90 (0.86–0.95)	0.92 (0.87–0.98)
**Continuity of care**			
University (ref.)	1	1	1
Apprentice/diploma	1.07 (1.03-1.11)	1.07 (1.02-1.11)	1.06 (1.01-1.11)
School certificate	1.11 (1.07-1.15)	1.18 (1.14-1.23)	1.18 (1.12-1.24)
No school certificate	1.14 (1.07-1.20)	1.24 (1.18-1.31)	1.15 (1.08-1.22)
**Care planning**			
University (ref.)	1	1	1
Apprentice/diploma	1.21 (1.14-1.29)	1.27 (1.19-1.35)	1.25 (1.15-1.36)
School certificate	1.32 (1.24-1.40)	1.40 (1.31-1.49)	1.43 (1.32-1.55)
No school certificate	1.53 (1.42-1.64)	1.64 (1.52-1.76)	1.61 (1.47-1.77)

Notes:
1. GP, general practitioner; OR, odds ratio; CI, confidence interval.
2. Adjusted for sociodemographic (educational attainment, age, sex, country of birth, marital status) and need (self-rated health status, number of chronic disease, physical functioning) variables.
3. Wald joint test of significance for education p<.001 for all outcomes and regions, except long consults in major cities (p<.05).

Supplementary table 7. Model 2: fixed effects for specialist use by region

Model 2, specialist use	Cities<br>OR (95%CI)	Inner regional<br>OR (95%CI)	Outer regional/ remote<br>OR (95%CI)
University (ref.)	1	1	1
Apprentice/diploma	0.94(0.91–0.98)	0.92(0.89–0.96)	0.95(0.91–1.00)
School certificate	0.90(0.86–0.93)	0.91(0.88–0.95)	0.95(0.91–1.00)
No school certificate	0.86(0.81–0.90)	0.85(0.81–0.90)	0.95(0.90–1.01)
p-value for SEP term	<.001	<.001	ns

Notes:
1. OR, odds ratio; CI, confidence interval; SEP, socioeconomic position; ns, not statistically significant.
2. Adjusted for sociodemographic (educational attainment, age, sex, country of birth, marital status) and need (self-rated health status, number of chronic disease, physical functioning) variables.
3. Wald joint test of significance for education cities and inner regional <.001, outer regional not significant.

For peer review only

STROBE Statement—Checklist of items that should be included in reports of ***cohort studies***

	Item No	Recommendation	Page No
**Title and abstract**	1	(*a*) Indicate the study's design with a commonly used term in the title or the abstract	1
		(*b*) Provide in the abstract an informative and balanced summary of what was done and what was found	2
**Introduction**			
Background/rationale	2	Explain the scientific background and rationale for the investigation being reported	4
Objectives	3	State specific objectives, including any prespecified hypotheses	4
**Methods**			
Study design	4	Present key elements of study design early in the paper	5
Setting	5	Describe the setting, locations, and relevant dates, including periods of recruitment, exposure, follow-up, and data collection	5
Participants	6	(*a*) Give the eligibility criteria, and the sources and methods of selection of participants. Describe methods of follow-up	5
		(*b*) For matched studies, give matching criteria and number of exposed and unexposed	na
Variables	7	Clearly define all outcomes, exposures, predictors, potential confounders, and effect modifiers. Give diagnostic criteria, if applicable	6
Data sources/ measurement	8*	For each variable of interest, give sources of data and details of methods of assessment (measurement). Describe comparability of assessment methods if there is more than one group	5 and 6
Bias	9	Describe any efforts to address potential sources of bias	7
Study size	10	Explain how the study size was arrived at	7
Quantitative variables	11	Explain how quantitative variables were handled in the analyses. If applicable, describe which groupings were chosen and why	6-7
Statistical methods	12	(*a*) Describe all statistical methods, including those used to control for confounding	7
		(*b*) Describe any methods used to examine subgroups and interactions	7
		(*c*) Explain how missing data were addressed	6
		(*d*) If applicable, explain how loss to follow-up was addressed	na
		(*e*) Describe any sensitivity analyses	7
**Results**			
Participants	13*	(a) Report numbers of individuals at each stage of study—eg numbers potentially eligible, examined for eligibility, confirmed eligible, included in the study, completing follow-up, and analysed	8
		(b) Give reasons for non-participation at each stage	8
		(c) Consider use of a flow diagram	
Descriptive data	14*	(a) Give characteristics of study participants (eg demographic, clinical, social) and information on exposures and potential confounders	8-9
		(b) Indicate number of participants with missing data for each variable of interest	Table 1
		(c) Summarise follow-up time (eg, average and total amount)	8
Outcome data	15*	Report numbers of outcome events or summary measures over time	Table 1

For peer review only - http://bmjopen.bmj.com/site/about/guidelines.xhtml

For peer review only

Main results	16	(*a*) Give unadjusted estimates and, if applicable, confounder-adjusted estimates and their precision (eg, 95% confidence interval). Make clear which confounders were adjusted for and why they were included	Figure 2, supplementary tables 3-7
		(*b*) Report category boundaries when continuous variables were categorized	
		(*c*) If relevant, consider translating estimates of relative risk into absolute risk for a meaningful time period	na
Other analyses	17	Report other analyses done—eg analyses of subgroups and interactions, and sensitivity analyses	10
**Discussion**			
Key results	18	Summarise key results with reference to study objectives	10
Limitations	19	Discuss limitations of the study, taking into account sources of potential bias or imprecision. Discuss both direction and magnitude of any potential bias	12
Interpretation	20	Give a cautious overall interpretation of results considering objectives, limitations, multiplicity of analyses, results from similar studies, and other relevant evidence	10-12
Generalisability	21	Discuss the generalisability (external validity) of the study results	12
**Other information**			
Funding	22	Give the source of funding and the role of the funders for the present study and, if applicable, for the original study on which the present article is based	15

*Give information separately for exposed and unexposed groups.

Note: An Explanation and Elaboration article discusses each checklist item and gives methodological background and published examples of transparent reporting. The STROBE checklist is best used in conjunction with this article (freely available on the Web sites of PLoS Medicine at http://www.plosmedicine.org/, Annals of Internal Medicine at http://www.annals.org/, and Epidemiology at http://www.epidem.com/). Information on the STROBE Initiative is available at http://www.strobe-statement.org.

For peer review only

BMJ Open

Does use of GP and specialist services vary across areas and according to individual-socioeconomic position? A multilevel analysis using linked data in Australia.

Journal:	*BMJ Open*
Manuscript ID	bmjopen-2023-074624.R1
Article Type:	Original research
Date Submitted by the Author:	07-Nov-2023
Complete List of Authors:	Butler, Danielle; Australian National University<br>Larkins, Sarah; James Cook University Division of Tropical Health and Medicine<br>Jorm, Louisa; University of New South Wales<br>Korda, Rosemary; Australian National University
**Primary Subject Heading**:	Health services research
Secondary Subject Heading:	General practice / Family practice, Health policy
Keywords:	Primary Health Care, Health Equity, Health policy < HEALTH SERVICES ADMINISTRATION & MANAGEMENT, EPIDEMIOLOGIC STUDIES

SCHOLARONE™
Manuscripts

I, the Submitting Author has the right to grant and does grant on behalf of all authors of the Work (as defined in the below author licence), an exclusive licence and/or a non-exclusive licence for contributions from authors who are: i) UK Crown employees; ii) where BMJ has agreed a CC-BY licence shall apply, and/or iii) in accordance with the terms applicable for US Federal Government officers or employees acting as part of their official duties; on a worldwide, perpetual, irrevocable, royalty-free basis to BMJ Publishing Group Ltd ("BMJ") its licensees and where the relevant Journal is co-owned by BMJ to the co-owners of the Journal, to publish the Work in this journal and any other BMJ products and to exploit all rights, as set out in our licence.

The Submitting Author accepts and understands that any supply made under these terms is made by BMJ to the Submitting Author unless you are acting as an employee on behalf of your employer or a postgraduate student of an affiliated institution which is paying any applicable article publishing charge ("APC") for Open Access articles. Where the Submitting Author wishes to make the Work available on an Open Access basis (and intends to pay the relevant APC), the terms of reuse of such Open Access shall be governed by a Creative Commons licence – details of these licences and which Creative Commons licence will apply to this Work are set out in our licence referred to above.

Other than as permitted in any relevant BMJ Author's Self Archiving Policies, I confirm this Work has not been accepted for publication elsewhere, is not being considered for publication elsewhere and does not duplicate material already published. I confirm all authors consent to publication of this Work and authorise the granting of this licence.

For peer review only

Does use of GP and specialist services vary across areas and according to individual-socioeconomic position? A multilevel analysis using linked data in Australia.

Author list

*Danielle C Butler[1], MBBS, MPH, FRACGP, PhD; ORID: 0000-0003-4870-4544

Sarah Larkins[2], MBBS, MPH&TM, FRACGP, PhD; ORCID: 0000-0002-7561-3202

Louisa R Jorm[3], BVSc, MSc. PhD, FAHMS, ORCID: 0000-0003-0390-661X

Rosemary J Korda[1], MAppSc, GradDipPopHlth, PhD, ORID: 0000-0002-9390-2171

Affiliations

1. National Centre for Epidemiology and Population Health, Australian National University
2. College of Medicine and Dentistry, James Cook University
3. Centre for Big Data Research in Health, University of New South Wales

***Corresponding author details**

Email: Danielle.Butler@anu.edu.au

Postal address: Building 62, Mills Rd, The Australian National University, Acton, ACT, Australia, 2601

Word count: 3652

Number of tables: 1

Number of Figures: 2

For peer review only - http://bmjopen.bmj.com/site/about/guidelines.xhtml

For peer review only

Abstract

Objective: Timely access to primary care and supporting specialist care relative to need is essential for health equity. However, use of services can vary according to an individuals' socioeconomic circumstances or where they live. This study aimed to quantify individual socioeconomic variation in GP and specialist use in New South Wales (NSW), accounting for area-level variation in use.

Design: Outcomes were GP use and quality-of-care and specialist use. Multilevel logistic regression was used to estimate: i)median odds ratios (MORs) to quantify small-area variation in outcomes, which gives the median increased risk of moving to an area of higher risk of an outcome, and ii) ORs to quantify associations between outcomes and individual education-level, our main exposure variable. Analyses were adjusted for individual sociodemographic and health characteristics and performed separately by remoteness categories.

Setting: Baseline data (2006–2009) from the 45 and Up Study, NSW, Australia, linked to Medicare Benefits Schedule (MBS) and death data (to December 2012).

Participants: 267, 112 adults aged 45 years and older.

Results: GP (MOR=1.32-1.35) and specialist use (1.16-1.18) varied between areas, accounting for individual characteristics. For a given level of need and accounting for area-variation, low education-level individuals were more likely to be frequent users of GP services (no school certificate vs university, OR=1.63-1.91, depending on remoteness category) and have continuity of care (OR=1.14-1.24), but were less likely to see a specialist (OR=0.85-0.95).

Conclusion: GP and specialist use varied across small-areas in NSW, independent of individual characteristics. Use of GP care was equitable, but specialist care was not. Failure to address inequitable specialist use may undermine equity gains within the primary care system. Policies should also focus on local variation.

Key words: primary care, equity, variation in care, socioeconomic inequalities, multilevel analysis

For peer review only - http://bmjopen.bmj.com/site/about/guidelines.xhtml

Strengths and limitations

- Our study used a multilevel analytical design which allowed quantification of area- and individual-level variation in primary care and supporting specialist care; quantifying these differing sources of variation is required for ensuring an equitable healthcare system.
- The large sample linked to health utilisation data, allowed quantification of observed use (rather than self-report) after accounting for a range of factors.
- The study sample was limited to those aged 45 year and older from Australia's most populous state and patterns of association may differ for other age groups or in other settings.

For peer review only - http://bmjopen.bmj.com/site/about/guidelines.xhtml

Introduction

Adequate and timely access to primary care relative to need is a specified goal of high performing health systems (1-3). This is integral to improving average levels of population health, as well as health equity (4). Further, an effective primary care system requires ready access to supporting specialist care. Yet often individuals' socioeconomic circumstances or where they live, as much as their need for care, determine their use of services (5-8); that is, access to care is inequitable. Examining and quantifying these differing sources of variation in care is essential for directing policy responses for achieving an equitable healthcare system.

There is evidence internationally (9, 10), and to a lesser extent within Australia (7, 8, 11-13), of socioeconomic variation in use of GP and specialist services. Across most jurisdictions, people who are of low socioeconomic position (SEP) use equal or more GP services for a given level of need relative to their high-SEP counterparts (7-11, 13). On the other hand, individuals of high-SEP are more likely to see a specialist than those of low-SEP (8-13). Use of primary care and specialist services also varies geographically. Studies in Australia using aggregated area-level data consistently find increased use of GP and specialist services in major cities compared with more remote areas (5, 12, 14). To date, no Australian studies have examined individual socioeconomic variation in use of primary and specialist services while accounting for area-variation in use of services, or quantified the extent of variation at the area-level, beyond that explained by the characteristics (such as sociodemographics or health status) of individuals living in those areas.

The aim of this study was to use large-scale linked data and multi-level analysis (15, 16) to examine the extent to which GP and specialist service use varied at the area-level, having accounted for the sociodemographic and health characteristics of people who lived in those areas. Further, we quantified variation in use of services according to individual SEP, having accounted for variation in use across areas. In this way, sources of variation in use of GP and specialist services are clarified and indicate directions for reducing unwarranted variation in care.

For peer review only - http://bmjopen.bmj.com/site/about/guidelines.xhtml

For peer review only

Methods

Study population and setting

The Sax Institute's 45 and Up Study is a large prospective cohort study involving 267,153 people aged 45 years and older residing in New South Wales, the most populous state in Australia (17). Participants were randomly sampled from the Services Australia (formerly the Australian Government Department of Human Services) Medicare enrolment database, with over-sampling by a factor of two of individuals aged 80 years and over and people resident in rural areas. Participants enrolled in the study by completing a baseline questionnaire, distributed between 2006 and 2009, and providing consent for 5-yearly questionnaires and linkage to routinely collected health data. About 19% of those invited participated in the study and participants included ~11% of the total NSW population aged 45 years and older (18). The study design and details of the questionnaire are reported elsewhere (18).

Data

Sociodemographic and health variables were derived from the self-reported baseline questionnaire. Data from the questionnaire were linked to Medicare Benefits Schedule (MBS) claims data (1 January 2003 – 14 December 2012) provided by Services Australia, and data from the NSW Registry of Births, Deaths and Marriages (RBDM) and the National Death Index (NDI). The MBS claims database includes all claims for subsidised medical and diagnostic services provided by registered medical and other practitioners through the MBS. For each claim for service processed, the MBS data include a range of information, including the date of the service and the item number for the service. Linkage of baseline data from 45 and Up Study participants to MBS data was performed at the Sax Institute through deterministic linkage, using an encrypted version of the Medicare number provided directly by Services Australia.

Probabilistic linkage to NSW RBDM was performed by the Centre for Health Record Linkage (CHeReL) data. Quality assurance data on the CHeReL data linkage show false positive and negative rates of <0.5% and <0.1%, respectively (19).

For peer review only - http://bmjopen.bmj.com/site/about/guidelines.xhtml

109 **Variables**

110 <u>Outcomes of interest</u>

111 For use of GP services, the main outcome was above-average GP use (no/yes) as a measure of frequent
112 use, defined as eight or more services in the year following completion of the baseline survey, which
113 is broadly consistent with definitions reported in the literature (8, 20). We also examined secondary
114 outcomes relating to types and qualities of GP services that indicate high-quality primary care (1-3),
115 and that the general population would be eligible to receive. This included: i) any MBS service for a
116 long or prolonged consultation (no/yes) in the follow up period (known to be associated with more
117 problems managed and better outcomes, (21, 22)); ii) continuity of GP care measured by the usual
118 provider continuity index (UPI) (23), calculated as the proportion of GP MBS services with the most
119 frequent provider of total GP MBS services and defined as a UPI of 70% or more. As per standard
120 definitions, the UPI was calculated over a 2-year period and calculated only for those participants who
121 used at least four GP services in that time; and iii) care planning (no/yes) defined as at least one MBS
122 service for a chronic disease and complex care planning item (including a GP management plan, team
123 care arrangement or review item) in the follow - up period. These items relate to specific MBS funded
124 services that can be claimed for care planning relating to chronic and complex care needs and to
125 enable multidisciplinary coordination of care.

126 Specialist use was defined as any out-of-hospital MBS specialist service in the follow up period
127 (no/yes). See additional file 1, supplementary Table 1 and 2 for full list of MBS items codes included in
128 the outcome measures.

129 <u>Main exposure variable</u>

130 Individual-level sociodemographic and health characteristics were derived from the 45 and Up
131 baseline questionnaire. Our main exposure variable, SEP, was measured as the highest educational
132 level attained (no school certificate, school certificate, apprenticeship or diploma, and university
133 degree).

For peer review only - http://bmjopen.bmj.com/site/about/guidelines.xhtml

Determinants of healthcare need and confounders

SEP is associated with morbidity and health status (24, 25), which in turn determines the need for healthcare. To determine need-adjusted use, healthcare need (8, 26) variables included were: self-reported health (excellent, very good, good, fair and poor); physical functioning (no limitation, minor limitation, moderate limitation, severe limitation and a missing category); and number of chronic conditions for the following self-reported conditions – cancer, asthma, hayfever, heart disease, heart attack, angina, stroke, diabetes, hypertension, hypercholesterolaemia, arthritis, osteoporosis, anxiety, depression and Parkinson's disease (none, 1-2 chronic conditions, 3 or more chronic conditions).

To account for confounders in the relationship between SEP or healthcare need and use of health services we also included: age (10 age categories from 45 years through to 85 years and over), sex (male/female); country of birth (Australia/NZ, Europe/Nth America, Asia, Africa/Mid East, and other); and marital status (married/defacto or not married/not defacto).

Using Australian Bureau of Statistics (ABS) concordance files, each participant was assigned to a Statistical Area Level 3 (SA3) geography. These areas have populations of between 30,000 and 130,000 persons and are considered representative of communities sharing similar characteristics in terms of services available.

Analysis

Participants were followed for one year after completing the baseline survey (most had completed this by 2008), and for two-years for continuity of care. We included participants if they had a least one Medicare record, were alive at the end of the follow up period, had a geographical identifier coded to NSW, and, for analyses of continuity of care, had at least 4 visits in the follow up period, and for analyses of care planning, had at least one long-term health condition.

Frequencies and proportions were calculated for the sample according to participant characteristics, for the total sample, by education and by outcomes. A two-level random intercept multilevel logistic regression model (participants nested within SA3 of residence) were fitted for each outcome. To

For peer review only - http://bmjopen.bmj.com/site/about/guidelines.xhtml

determine if there was significant area-level variation in outcomes, and hence a random intercept model appropriate, a model without explanatory variables (null model) was first fitted (additional file 1, supplementary Tables 3-5). For the main analysis, the model was adjusted for individual education, healthcare need and confounders to determine need-adjusted individual-level socioeconomic variation in outcomes (having accounted for area-level variation).

Area-level variation in each outcome was estimated from the variance term (V_A) by calculating the ICC by the linear threshold model method ($ICC=V_A/(V_A+3.29)$ and the median odds ratio ($MOR=\exp(0.954\sqrt{V_A})$ (15). Conceptually, the MOR is the median of the odds ratios calculated from pairwise comparison of people with identical covariates, but residing in different areas. It quantifies the median increased risk that would occur if moving from one area to another with higher risk(15). The proportional change in variance ($PCV=(V_A-V_B/V_A) \times 100$) (15) was used to estimate the proportion of overall variation in outcome explained by addition of explanatory variables to the model. Second-order penalised quasi-likelihood (PQL) estimation was used as per Rasbash and colleagues (16). Monte Carlo Markov Chain (MCMC) estimation was used to assess model fit statistics and residuals plotted to test model assumptions held.

As health service use in Australia varies according to remoteness, analyses were stratified by categories of remoteness (major cities, inner regional, outer regional/remote) based on the 2006 Access and Remoteness Index of Australia (+) (27) and according to the Australian Statistical Geography Standard Classification of remoteness (ASGC-RA).

Analyses were undertaken using Stata (College Station, Texas, StataCorp; Version 14.1) in the Secure Unified Research Environment, a secure remote-access computer facility for analysis of linked data. Multilevel analysis were performed using the runmlwin add-on (28), using Stata's post estimation procedures.

Sensitivity analyses were also repeated using alternative measures of frequency of GP use (low versus medium and medium versus high) and including those who died in the follow up period.

For peer review only

For peer review only - http://bmjopen.bmj.com/site/about/guidelines.xhtml

Ethics approval for this project was obtained from the NSW Population and Health Services Research Ethics Committee (HREC/13/CIPHS/8), the University of Western Sydney Ethics Committee (H9835) and the Australian National University Human Research Ethics Committee (2011/703). Ethics approval for the 45 and Up Study was granted by the University of New South Wales Human Research Ethics Committee. The 45 and Up Study participants consented to data linkage at baseline. Linkage of the MBS data is performed under approvals from the ethics committees of Services Australia and the Australian Government Department of Health.

Patient and public involvement

No patients or members of the public were involved in the design or conduct of the study.

Results

Sample Characteristics

After excluding those who had an invalid death date or died in the follow up period (n=320), did not have an MBS service (n=1583), or were unable to be assigned to an SA3 (n=151) the final sample for inclusion was 263,083. Of these, 11.7% had no school certificate, 31.8% completed a school certificate, 31.8% had completed an apprenticeship or diploma and 23% had completed a tertiary level qualification. The mean age of the population was 62.7 years (SD 11.2), 46% were male, over 80% rated their health as good, very good or excellent and 73% had at least one chronic condition (Table 1).

[Table 1 here]

Area-level variation

Use of GP services varied according to where a person resided–for all regions–having accounted for the characteristics of individuals living in those areas (Figure 1, MOR major cities 1.34, inner regional 1.32, outer regional/remote 1.35). This means that an individual who lived in area with a higher rate of above-average GP use had a (median) 32-34% greater probability of having above-average GP use

For peer review only - http://bmjopen.bmj.com/site/about/guidelines.xhtml

than an individual with identical characteristics who lived in an area with a lower rate of above-average GP use. Quality of GP care similarly varied across areas (additional file 1, supplementary Table 4). Area-level variation in specialist use across all regions was also evident after accounting for the characteristics of individuals (MOR 1.16–1.18; additional file 1, supplementary Table 5).

[Figure 1 here]

Individual level socioeconomic variation

People of low education-level used more GP services on average compared to those with higher levels of education, for a given level of need and having accounted for area variation in use (Figure 2). For secondary outcomes examining quality of GP care, people of low education-level were also more likely to have care planning (e.g. no school certificate vs university educated in major cities 1.53[1.42, 1.14]) and continuity of care (e.g. in major cities 1.14[1.07, 1.20]) compared to their high education-level counterparts, but less likely to have a long consultation (e.g. inner regional 0.90[0.87, 0.95]), accounting for area-variation in these outcomes (additional file 1, supplementary Table 6). Patterns of association were found whether in major cities or more remote locations.

[Figure 2 here]

On the other hand, people of low education-level (for a given level of need) were less likely have a specialist service compared to their higher-education counterparts, accounting for area-variation (Figure 2, additional file 1, supplementary Table 7).

Sensitivity analyses did not differ materially from the main findings.

Discussion

This study has shown, that where people live (at the local area-level) matters for the GP and specialist services they receive, independent of their personal characteristics. This was the case across all remoteness categories - major cities, regional and more remote areas - in New South Wales, Australia. Further, having accounted for where people live, use of GP services and quality of care was equitable,

For peer review only - http://bmjopen.bmj.com/site/about/guidelines.xhtml

For peer review only

in that for a given level of need disadvantaged people were more likely to use more services on average, and to have continuity of care and care planning. However, the finding that advantaged people were more likely to see a specialist or have a long consultation suggests a potential source of inequity.

This is the first study in Australia and one of few internationally to quantify area-level variation in GP and specialist use, independent of the characteristics of people who lived in these areas. The amount of variation between areas quantified in this study is comparable to that previously reported when examining other healthcare outcomes in Australia (e.g. hospitalisations (29)), and internationally (30). More use of GP services and care planning and greater continuity of care among people of lower SEP has been previously shown in Australia (7, 8, 11, 13, 31, 32) and internationally (10, 33, 34). This study confirms that this holds having accounted for where people live. Previous studies have demonstrated inequity of specialist use (10, 11). However, only one study accounted for where people live and this was in a country without gate-keeping mechanisms in place where a person can consult a specialist without referral from a GP or primary care physician(30). Our study further confirms this finding in a context of gate-keeping policies.

We found that individual use and quality of GP and specialist services varied across small-areas, for all remoteness categories, beyond what could be explained by the characteristics of people living in those areas. This suggests that there are aspects within peoples' local context that systematically shape the care of all who live in that area. The specific reasons are unknown but may relate to how services are organised and delivered (including availability of providers) within an area or structural policies determining the geographical distribution of services and providers. International multilevel studies in countries with (35) and without (30) a gate-keeping mechanism have shown that availability of GPs and specialists within an area was associated with specialist use. This has not been investigated for GP service use or quality of care. Importantly, how services are organised can be changed (through policy and practice) and doing so may contribute to reducing the unwarranted variation across areas.

For peer review only

Our finding of more care planning and greater continuity of care among people with lower levels of education highlights an important source of equity in the Australian primary healthcare system. People of lower SEP are more likely to have multiple and complex health and psychosocial care needs (24, 36) than their advantaged counterparts, and continuity of care and care planning are essential for enabling these needs to be met. In Australia, practices and/or clinicians may provide Medicare subsided services without the patient making an additional co-payment (referred to as bulk-billed). A possible explanation for the association with continuity of care observed here, is that disadvantaged people are more likely to attend such services, thereby limiting the number of providers available to them. Future research exploring this will be informative for understanding the mechanism underlying this relationship.

There are likely multiple reasons why socioeconomically disadvantaged people use less specialist services for a given level of need. In Australia, GPs are incentivised to bulk-bill specific population groups. However, these are unavailable for specialists. Out-of-pocket costs for specialist services doubled in the decade prior to the study period (37) and have continued to rise since. Further, private health insurance has been shown to contribute to pro-high income use of specialist services (8, 12); yet government funded rebates for private health insurance have remained in place. Other possible reasons include: differences in propensity to seek care due to differences in health literacy, attitudes and beliefs; or, due to negatively biased behaviours from providers, disadvantaged people are less likely to seek specialist care (38). However, if this was the case a similar finding would be expected with use of GP services. Further, studies examining propensity to seek care (39) or rates of completion of specialist referrals(40) have not found differences between socioeconomic groups.

Alternatively, these differences may be due to provider preferences and bias. International evidence also suggests providers offer fewer services to those of low SEP (38) and are more likely to refer higher SEP individuals to a specialist (41). Irrespective of the reasons, differences in use does not reflect need for care and hence is inequitable and unjust.

For peer review only

The reason why people of high education were more likely to have a long consultation is unknown. Possibly, people with lower educational attainment are more likely to be bulk-billed, and given current financing arrangements in Australia, the benefit per minute falls with longer consultations. These findings may also reflect differences in health literacy. People with higher levels of education may be more likely to anticipate and expect a range a health issues to be addressed in a single episode and request a consultation length to that effect, or actively seek out practitioners with characteristics associated with longer consultations(42).

A strength of our study is the multilevel analytical design, which allowed modelling of nested levels of data and quantification of area- and individual-level variation. Further, the large sample linked to MBS service use, allowed quantification of observed use (rather than self-report) after accounting for a range of factors. While MBS data will capture nearly all GP services, there are some settings where services provided do not attract an MBS claim. For example, publicly funded community health centres and some GP services provided in emergency departments in rural and remote areas. In addition, a substantial proportion of specialist services in Australia are provided in publicly funded hospital-based outpatient clinics, which generally do not attract an MBS rebate. Low-SEP people are more likely to use these community and hospital-based services(8) and exclusion of these services may bias estimates for SEP gradients to be pro-high SEP. However, previous studies found this did not alter estimates of socioeconomic variation in GP and ambulatory specialist care(8). The 45 and Up study is not representative of the NSW population (43), and while representativeness is not necessary for internal validity (i.e. relative effect estimates) (43), patterns of association may differ for other age groups or in other settings.

Implications for policy and practice.

An effective PC system requires ready and reliable access to secondary level care. This has not been equitably achieved in Australia–despite the presence of universal health insurance–undermining the equity that exists in the PC system. National structural policies, such as minimising out-of-pocket costs

For peer review only

for example through bulk-billing incentives, would go some way to redressing inequitable use of specialist services. Given that private health insurance contributes to pro-high SEP use of specialist services(13), offsetting government rebates in favour of lower income or disadvantaged individuals could also contribute to reducing this inequity. It could be argued that the inequity in community-based specialist services is balanced by a pro-low SEP preference for specialist outpatient services through the public hospital sector. However, waiting times for less urgent and more discretionary health needs (and in some instances for more urgent health needs) in the public sector are understood to exceed that in the private sector (44), although actual wait times are not published. This increases the impact of illness on recovery and quality of life, affecting those who are disadvantaged to a greater extent. As such, addressing inequalities in access to specialist care is even more pressing.

The unwarranted variation in both GP and specialist use at the area-level suggests that additional policy approaches are needed that are directed to local contexts, in addition to individuals. For example, it may be that availability of providers (both GPs and specialists) may need to be addressed, as international studies have shown this explains some of the area-level variation in care. Similarly, there may be other aspects of how services are organised and delivered at the local area-level that may determine peoples' use of services. The specific drivers and hence policy solutions to addressing the unwarranted area-level variation requires further exploration.

Conclusion

It is reassuring that, for a given level of need, GP service use and important aspects of quality of care (such as care planning, continuity of care) favours those who are disadvantaged; further, this is the case regardless of where people live. However, the ongoing pro-high SEP use of specialist service threatens to undermine this and requires urgent attention. Equity measures to improve affordability are an important avenue to address this. However, both GP and specialist care varies not only between major cities and more remote locations, but also within at the small area-level. This is unwarranted and highlights an important opportunity to improve equity in the Australian healthcare system.

For peer review only - http://bmjopen.bmj.com/site/about/guidelines.xhtml

Table 1. Sample characteristics: individual-level variables by educational attainment (%) and for total sample

Variable	*Educational attainment*					
	No school certificate	School certificate	Apprentice/ diploma	University	Missing	Row category total %(n)
Education						
Total %(n)	11.7(31,126)	31.8(84,302)	31.8(84,294)	23(60,933)	1.7(4,428)	100(265,083)
Sex						
Male	42.1	36.1	55.2	50.0	48.6	46(122,893)
Female	57.3	63.9	44.8	50.0	51.5	53.6(142,190)
Age						
45–54	16.5	24.4	31.6	40.1	14.4	29.2(77,397)
55–64	27.4	32.9	31.9	34.8	22.6	32.2(85,342)
65–74	28.9	23.7	21.5	15.7	25.4	21.8(57,734)
75–84	21.8	15.3	12.7	8.0	28.8	13.8(36,516)
85 plus	5.4	3.8	2.3	1.5	8.8	3.1(8,082)
Country of birth						
Australia/NZ	75.9	80.8	76.9	72.5	64.8	76.8(203,629)
Europe/ N. America	18.6	13.4	17.7	16.8	20.9	16.3(43,154)
Asia	2.3	2.6	2.3	6.5	4.0	3.4(9,031)
Africa/Mid. East	1.1	1.5	1.3	2.7	1.7	1.7(4,397)
Other	0.5	0.8	0.9	0.8	0.9	0.08(2,145)
Marital status						
Not married/ not de facto	32.6	26.2	22.3	21.0	33.5	24.7(65,288)
Married/de facto	66.8	73.3	77.1	78.5	64.3	74.7(198,185)
Self-rated health						
Excellent	7.4	12.2	14.1	22.4	10.1	14.6(38,575)
Very good	25.6	35.0	36.8	40.8	24.6	35.6(94,481)
Good	36.3	34.5	33.8	26.5	32.1	32.6(86,451)
Fair	20.5	12.3	10.7	6.9	17.5	11.6(30,644)
Poor	4.8	2.2	1.8	1.0	4.0	2.1(5,575)

For peer review only

Variable	Educational attainment					
	No school certificate	School certificate	Apprentice/ diploma	University	Missing	Row category total %(n)
Chronic conditions						
none	20.7	25.4	27.3	31.2	25.2	26.8(70,991)
1–2	49.9	52.0	52.6	53.0	50.1	52.1(198,116)
3 or more	29.4	22.6	20.2	15.8	24.7	21.1(55,976)
Physical functioning						
No limitation	18.9	26.5	30.0	39.4	19.2	29.5(78,323)
Minor limitation	15.4	23.2	26.9	30.2	14.5	24.9(66,072)
Moderate limitation	21.4	22.5	21.6	17.3	16.7	20.8(55,097)
Severe limitation	21.8	12.9	10.3	5.5	16.5	11.5(30,367)
GP use						
Below average	46.5	59.0	64.4	74.3	47.8	62.6(165,803)
Above average	53.6	41.1	35.6	25.7	52.2	37.5(99,280)
Continuity of care						
<70%	41.1	44.8	46.5	50.4	41.6	46.1(105,433)
≥70%	58.7	55.0	53.4	49.6	58.1	53.7(128,055)
Any care planning						
No	53.5	54.8	55.3	56.3	51.6	55.1(146,046)
Yes	22.4	15.9	13.5	8.5	20.1	14.3(37,815)
Any long consult						
No	56.5	58.7	60.1	59.8	56.2	59.1(156,652)
Yes	43.5	41.3	39.9	40.2	43.8	40.9(108,428)
Any specialist use						
No	40.5	44.3	46.5	47.9	40.5	45.3(120,063)
Yes	59.5	55.7	53.6	52.1	59.5	54.7(145,019)

Notes: N, number; %, percentage; Columns for each variable category for each educational attainment categories sum to 100%. Values in last column gives break down by category for each individual variables for the total sample, not stratified by educational attainment. For each variable, total (n) sums to 265,083 and percent sums to 100% including missing data;Chi-squared test for trend with education p<.001 all variables. Missing: age<1%, country of birth 1%, marital status 0.6%, self-rated health 3.5%, physical functioning 13.3%. For analyses of continuity of care, those who died in the second year or had less than four GP MBS services during follow-up were excluded (n=31595). Additional exclusions included: i) care planning participants without a chronic disease (n=81222); and ii) outlier observation of long consultations (n=3).

For peer review only - http://bmjopen.bmj.com/site/about/guidelines.xhtml

Figure titles and legends

Figure 1. Difference between mean for each area (SA3) and the mean across all areas in log odds of above-average use (95% CI) for each area, by remoteness

Notes: Adjusted for education, age, sex, country of birth, marital status, self-rated health, chronic disease and physical functional limitation; Mean log odds of above-average use across areas for that remoteness category set 0 and given by the horizontal red line; Each dot represents the mean for each SA3 of the difference in log odds of above-average use for each person in that SA3 from the mean log odds of above-average use for all areas (i.e. the mean of the residuals by SA3). Bars are the 95% confidence intervals around the mean for each SA3. SA3 values that lie above and below the red with CIs that do not cross the red line, are significantly different from the mean log odds of above-average use for all SA3s in that remoteness category. A person living in an area above the line has a higher probability of above-average GP use than the overall sample mean, irrespective of their individual characteristics; CI, confidence interval; SA3, statistical area 3.

Figure 2. Odds ratios and 95% confidence intervals for education with above-average use of GP services and any use of specialist services, by remoteness.

Notes: GP, general practitioner; OR, odds ratio; CI, confidence interval; %, percentage; ref., reference. Model fitted with a random intercept (area-level) adjusted for sociodemographic (education, age, sex, country of birth, marital status) and need (self-rated health status, number of chronic disease, physical functioning) variables. GP use, Wald joint test of significance for education p<.001 for all remoteness categories. Any specialist use Wald joint test of significance for education cities and inner regional <.001, outer regional not significant.

For peer review only

Supplementary Information

Additional file 1:

References

1. olde Hartman TC, Bazemore A, Etz R, Kassai R, Kidd M, Phillips RL, et al. Developing measures to capture the true value of primary care. BJGP Open. 2021:BJGPO.2020.0152.

2. Veillard J, Cowling K, Bitton A, Ratcliffe H, Kimball M, Barkley S, et al. Better Measurement for Performance Improvement in Low- and Middle-Income Countries: The Primary Health Care Performance Initiative (PHCPI) Experience of Conceptual Framework Development and Indicator Selection. Milbank Q. 2017;95(4):836-83.

3. Kringos DS, Boerma WG, Bourgueil Y, Cartier T, Hasvold T, Hutchinson A, et al. The European primary care monitor: structure, process and outcome indicators. BMC Fam Pract. 2010;11:81.

4. Solar O, Irwin A. A conceptual framework for action on the social determinants of health. Social Determinants of Health discussion paper 2 (policy and practice). Geneva: World Health Organisation; 2010.

For peer review only - http://bmjopen.bmj.com/site/about/guidelines.xhtml

5. Mu C, Hall J. What explains the regional variation in the use of general practitioners in Australia? BMC Health Services Research. 2020;20(1):325.

6. Haggerty J, Levesque J-F, Harris M, Scott C, Dahrouge S, Lewis V, et al. Does healthcare inequity reflect variations in peoples' abilities to access healthcare? Results from a multi-jurisdictional interventional study in two high-income countries. Intern. 2020;19(1):167.

7. Ou L, Chen J, Hillman K. Socio-demographic disparities in the utilisation of general practice services for Australian children - Results from a nationally representative longitudinal study. PloS one. 2017;12(4):e0176563.

8. Korda RJ, Banks E, Clements MS, Young AF. Is inequity undermining Australia's 'universal' health care system? Socio-economic inequalities in the use of specialist medical and non-medical ambulatory health care. Aust Nz J Publ Heal. 2009;33(5):458-65.

9. Devaux M, de Looper M. Income related inequalities in health service utilisation in 19 OECD countries, 2008-2009. OECD Publishing, Paris. http://dx.doi.org/10.1787/5k95xd6stnxt-en; 2012.

10. Lueckmann SL, Hoebel J, Roick J, Markert J, Spallek J, von dem Knesebeck O, et al. Socioeconomic inequalities in primary-care and specialist physician visits: a systematic review. Intern. 2021;20(1):58.

11. Dalziel KM, Huang L, Hiscock H, Clarke PM. Born equal? The distribution of government Medicare spending for children. Social Science & Medicine. 2018;208:50-4.

12. Pulok MH, van Gool K, Hall J. The link between out-of-pocket costs and inequality in specialist care in Australia. Australian Health Review. 2022;46(6):652-9.

13. Pulok MH, van Gool K, Hall J. Inequity in physician visits: the case of the unregulated fee market in Australia. Social Science & Medicine. 2020;255:113004.

14. AIHW. Medicare-subsided GP, allied health and specialist care across local areas: 2013-14 to 2018-19 Canberra2020 [7 August 2021]. Available from: https://www.aihw.gov.au/reports/primary-health-care/medicare-subsidised-health-local-areas-2019/contents/introduction.

For peer review only

For peer review only - http://bmjopen.bmj.com/site/about/guidelines.xhtml

15. Merlo J, Chaix B, Ohlsson H, Beckman A, Johnell K, Hjerpe P, et al. A brief conceptual tutorial of multilevel analysis in social epidemiology: using measures of clustering in multilevel logistic regression to investigate contextual phenomena. J Epidemiol Community Health. 2006;60(4):290-7.

16. Rasbash J, Steel BJ, Browne WJ, Goldstein H. A user's guide to MLwin. University of Bristol: Centre for Multilevel Modelling; 2012.

17. ABS. 2071.0 Census of population and housing: Reflecting Australia-stories from the Census, 2016. Canberra: ABS; 2016.

18. Banks E, Redman S, Jorm L, Armstrong B, Bauman A, Beard J, et al. Cohort profile: the 45 and up study. Int J Epidemiol. 2008;37(5):941-7.

19. NSW Centre for Health Record Linkage. 2018 [Available from: http://www.cherel.org.au/.

20. Smits FTM, Mohrs JJ, Beem EE, Bindels PJE, van Weert HC. Defining frequent attendance in general practice. BMC Fam Pract. 2008;9.

21. Wilson A, Childs S. The relationship between consultation length, process and outcomes in general practice: a systematic review. Br J Gen Pract. 2002;52(485):1012-20.

22. Britt HC, Valenti L, Miller GC. Determinants of consultation length in Australian general practice. Med J Aust. 2005;183(2):68-71.

23. Jee SH, Cabana MD. Indices for continuity of care: a systematic review of the literature. Medical care research and review : MCRR. 2006;63(2):158-88.

24. Dugravot A, Fayosse A, Dumurgier J, Bouillon K, Rayana TB, Schnitzler A, et al. Social inequalities in multimorbidity, frailty, disability, and transitions to mortality: a 24-year follow-up of the Whitehall II cohort study. The Lancet Public Health. 2020;5(1):e42-e50.

25. Korda RJ, Paige E, Yiengprugsawan V, Latz I, Friel S. Income-related inequalities in chronic conditions, physical functioning and psychological distress among older people in Australia: cross-sectional findings from the 45 and up study. BMC Public Health. 2014;14:741.

26. Andersen RM. Revisiting the behavioral model and access to medical care: does it matter? J Health Soc Behav. 1995;36(1):1-10.

For peer review only

For peer review only - http://bmjopen.bmj.com/site/about/guidelines.xhtml

27. Australian Bureau of Statistics. Remoteness Structure 2015 [Available from: http://www.abs.gov.au/websitedbs/d3310114.nsf/home/remoteness+structure.

28. Leckie G, Charlton C. runmlwin: A program to run the MLwiN multilevel modeling software from within Stata. Journal of Statistical Software. 2012;52(11):1-40.

29. Falster MO, Jorm LR, Douglas KA, Blyth FM, Elliott RF, Leyland AH. Sociodemographic and health characteristics, rather than primary care supply, are major drivers of geographic variation in preventable hospitalizations in Australia. Med Care. 2015;53(5):436-45.

30. Chaix B, Boelle PY, Guilbert P, Chauvin P. Area-level determinants of specialty care utilization in France: a multilevel analysis. Public Health. 2005;119(2):97-104.

31. Douglas KA, Yen LE, Korda RJ, Kljakovic M, Glasgow NJ. Chronic disease management items in general practice: a population-based study of variation in claims by claimant characteristics. Med J Australia. 2011;195(4):198-202.

32. McRae I, Yen L, Gillespie J, Douglas K. Patient affiliation with GPs in Australia--who is and who is not and does it matter? Health Policy. 2011;103(1):16-23.

33. Kristjansson E, Hogg W, Dahrouge S, Tuna M, Mayo-Bruinsma L, Gebremichael G. Predictors of relational continuity in primary care: patient, provider and practice factors. BMC Fam Pract. 2013;14:72.

34. Jatrana S, Crampton P, Richardson K. Continuity of care with general practitioners in New Zealand: results from SoFIE-Primary Care. The New Zealand medical journal. 2011;124(1329):16-25.

35. Chan BT, Austin PC. Patient, physician, and community factors affecting referrals to specialists in Ontario, Canada: a population-based, multi-level modelling approach. Med Care. 2003;41(4):500-11.

36. Stevenson AD, Phillips CB, Anderson KJ. Resilience among doctors who work in challenging areas: a qualitative study. Br J Gen Pract. 2011;61(588):e404-10.

For peer review only

For peer review only - http://bmjopen.bmj.com/site/about/guidelines.xhtml

37. Australian Government Department of Health. Annual medicare statistics financial year 1984-85 to 2015-16 2017 [Available from: http://www.health.gov.au/internet/main/publishing.nsf/content/annual-medicare-statistics.

38. Dovidio JF, Fiske ST. Under the Radar: How Unexamined Biases in Decision-Making Processes in Clinical Interactions Can Contribute to Health Care Disparities. American journal of public health. 2012;102(5):945-52.

39. van Loenen T, van den Berg MJ, Faber MJ, Westert GP. Propensity to seek healthcare in different healthcare systems: analysis of patient data in 34 countries. BMC Health Services Research. 2015;15(1):465.

40. Forrest CB, Shadmi E, Nutting PA, Starfield B. Specialty Referral Completion Among Primary Care Patients: Results From the ASPN Referral Study. Ann Fam Med. 2007;5(4):361-7.

41. Sørensen TH, Olsen KR, Vedsted P. Association between general practice referral rates and patients; socioeconomic status and access to specialised health care. Health Policy. 2009;92(2):180-6.

42. Wilson A, Childs S. The effect of interventions to alter the consultation length of family physicians: a systematic review. Br J Gen Pract. 2006;56(532):876-82.

43. Mealing NM, Banks E, Jorm LR, Steel DG, Clements MS, Rogers KD. Investigation of relative risk estimates from studies of the same population with contrasting response rates and designs. BMC Med Res Methodol. 2010;10:26.

44. Public-private time the key to reining in public hospital specialist wait lists [press release]. Univerity of Melbourne2016.

Declarations

Ethics approval and consent to participate

Ethics approval for this project was obtained from the NSW Population and Health Services Research Ethics Committee (HREC/13/CIPHS/8), the University of Western Sydney Ethics Committee (H9835)

For peer review only - http://bmjopen.bmj.com/site/about/guidelines.xhtml

and the Australian National University Human Research Ethics Committee (2011/703). Ethics approval for the 45 and Up Study was granted by the University of New South Wales Human Research Ethics Committee. The 45 and Up Study participants consented to data linkage at baseline. Linkage of the MBS data is performed under approvals from the ethics committees of Services Australia and the Australian Government Department of Health.

Consent for publication

Not applicable

Availability of data and materials

The data that support the findings of this study are available from the Sax Institute, NSW but restrictions apply to the availability of these data, which were used under license for the current study, and so are not publicly available. Data part of the Sax Institute's 45 and Up Study are available for approved projects to approved researchers (www.saxinstitute.org.au).

Competing interests

The authors declare that they have no competing interests.

Funding

This research was supported through a grant from the Australian Government through the National Health and Medical Research Council Postgraduate Scholarship (GNT1038903).

Authors' contributions

DB, LJ, SL, RK conceived and designed the analysis. DB completed data analysis and drafted the manuscript. All authors revised the work for intellectual content and approved the final version of the manuscript.

Acknowledgements

For peer review only

This research was completed using data collected through the 45 and Up Study (www.saxinstitute.org.au). The 45 and Up Study is managed by the Sax Institute in collaboration with major partner Cancer Council NSW; and partners: the Heart Foundation; NSW Ministry of Health; NSW Department of Communities and Justice; and Australian Red Cross Lifeblood. We thank the many thousands of people participating in the 45 and Up Study.

For peer review only

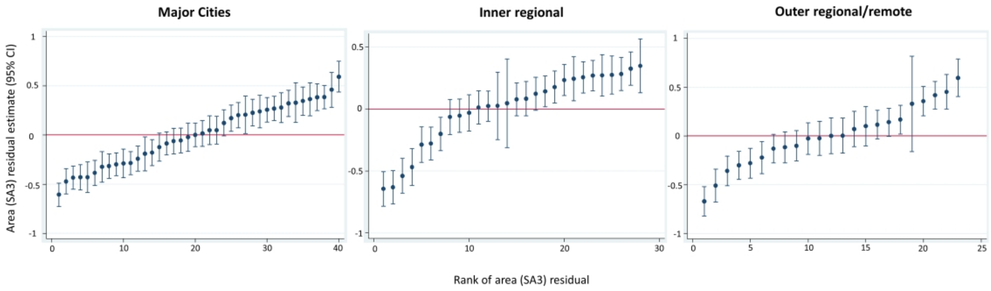

Figure 1. Difference between mean for each area (SA3) and the mean across all areas in log odds of above-average use (95% CI) for each area, by remoteness
Notes: Adjusted for education, age, sex, country of birth, marital status, self-rated health, chronic disease and physical functional limitation; Mean log odds of above-average use across areas for that remoteness category set 0 and given by the horizontal red line; Each dot represents the mean for each SA3 of the difference in log odds of above-average use for each person in that SA3 from the mean log odds of above-average use for all areas (i.e. the mean of the residuals by SA3). Bars are the 95% confidence intervals around the mean for each SA3. SA3 values that lie above and below the red with CIs that do not cross the red line, are significantly different from the mean log odds of above-average use for all SA3s in that remoteness category. A person living in an area above the line has a higher probability of above-average GP use than the overall sample mean, irrespective of their individual characteristics; CI, confidence interval; SA3, statistical area 3.

338x190mm (300 x 300 DPI)

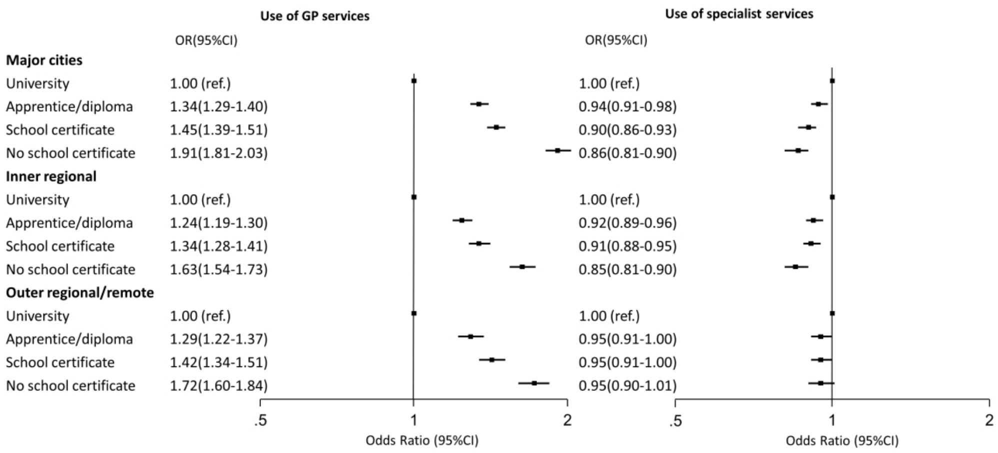

Figure 2. Odds ratios and 95% confidence intervals for education with above-average use of GP services and any use of specialist services, by remoteness.
Notes: GP, general practitioner; OR, odds ratio; CI, confidence interval; %, percentage; ref., reference. Model fitted with a random intercept (area-level) adjusted for sociodemographic (education, age, sex, country of birth, marital status) and need (self-rated health status, number of chronic disease, physical functioning) variables. GP use, Wald joint test of significance for education p<.001 for all remoteness categories. Any specialist use Wald joint test of significance for education cities and inner regional <.001, outer regional not significant.

338x190mm (300 x 300 DPI)

Title: Area-level and individual-socioeconomic variation in use of GP and specialist services. A multilevel analysis using linked data.

Supplementary table 1: Item numbers for GP services

MBS item groups and name of group	Specific item numbers within item group	Notes on item numbers
A1-GP attendances	3, 23, 36, 44<br><br>Remainder A1 attendances=4, 20, 24, 35, 37, 43, 47, 51	Items for services in consulting rooms<br>Items relate to consultations at residential care OR other than residential care/consulting rooms
A2-MPs other than GP	52,53,54,57<br><br>Remainder of A2 attendances=58–60, 65, 92, 93, 95, 96	Items for services in consulting rooms<br>Items relate to consultations at residential care OR other than residential care/consulting rooms
A11-GP	597, 599	Urgent after-hours care
A11-MP other than GP	598, 600	Urgent after-hours care
A14-heath Assessments	701, 703, 705, 707	
A14-health assessments for Aboriginal and Torres Strait Islander peoples	715	
A15-GP management plans	721, 723, 729, 731, 732	Care plans, care plan reviews and team care arrangements
A15-case conference	735, 739, 743, 747, 750, 758	
A17	900 and 903	Medication reviews
A18-GP, cervical screening	2497, 2501, 2503, 2504, 2506, 2507, 2509	Incentive for overdue pap smears (>4 years since last pap)
A18-GP asthma	2546, 2547, 2552, 2553, 2558, 2559	cycle of care
A18-GP diabetes	2517, 2518, 2521, 2522, 2525, 2526	cycle of care
A19-non-referred cervical screening	2598, 2600, 2603, 2606, 2610, 2613, 2616	As per A19 for non-VR MPs
A19-non-referred, asthma	2664, 2666, 2668, 2673, 2675, 2677	As for A18 for non-VR MPs
A19-non-referred, diabetes	2620, 2622, 2624, 2631, 2633, 2635	As for A18 for non-VR MPs
A20-GP mental health care plans	2700, 2701, 2712, 2713, 2715, 2717, 2721, 2723, 2725, 2727	Preparation and review of mental health care plans
A22-after hours (GP attendances)	5000, 5003, 5010, 5020, 5023, 5028, 5040, 5043, 5049, 5060, 5063, 5067	Includes consultation room, residential facility and other. At consultation rooms bolded
A23-after hours (non-referred)	5200, 5203, 5207, 5208, 5220, 5223, 5227, 5228, 5260, 5263, 5265, 5267	As per A22 for non-VR MPs

Notes:

1. GP, general practitioner; MP, medical practitioner; VR vocationally registered.
2. All item numbers were included for frequency of GP use and continuity of care outcome measures. The specific item numbers included for length of consultation were 36, 44, 54, and 57. The specific item numbers for care planning included all those listed under A15 GP management plans.
3. Medical practitioners (MPs) in this case refer to medical doctors who provide non-referred services but are not vocationally registered as a GP (i.e. have not undertaken specialist training in General Practice or other equivalent recognised training program).

For peer review only

Supplementary table 2. Item numbers included for specialist use

MBS item groups and name of group	Specific item numbers within item group	Notes on item numbers
A3-specialist	99, 104, 105, 106, 107, 108, 109, 113	includes home visits; visits at surgery or hospital 99; 113=telehealth
A4-consultant physician	110, 112, 114, 116, 119, 122, 128, 131, 132, 133	112 & 114 telehealth; 122=home visit
A28-geriatric medicine	141, 143,145, 147, 149	145=home visit; 149 telehealth
A8-consultant psychiatry	288, 291, 293, 296, 299, 300, 302, 304, 306, 308, 310, 312, 314, 316, 318, 319, 330, 332, 334, 336, 338, 342, 344, 346, 353, 355, 356, 357, 358, 359, 361, 364, 366, 367, 369, 370	288=telehealth; 289 not included, for autism developmental delay, referred from any practitioner; 299=home visit, hospital consults excluded, interviewing other than patient excluded; 353–370 telepsychiatry
A12-occupational physician	384, 385, 386, 387, 388, 389	
A13-public health	410, 411, 412, 413, 414, 415, 416, 417	Case conferences excluded
A24-pain and palliative medicine	2799, 2801, 2806, 2814, 2820, 2824, 2832, 2840, 3003, 3005, 3010, 3014, 3015, 3018, 3023, 3028	Case conferences excluded
A26-neurosurgery	6004, 6007, 6009, 6011, 6013, 6015, 6016	

1. Note:
2. A3-specialist also includes all attendances by surgeons (at surgery/consulting rooms or hospital). With respect to surgical specialists, only neurosurgery has specific item numbers for attendances.

Supplementary table 3. Between-area variation in odds of above-average GP use.

	Null model	Model 2
**Cities**		
ICC	0.044	0.028
PCV (%)	na	36
MOR	1.45	1.34
**Inner regional**		
ICC	0.023	0.026
PCV (%)	na	-13
MOR	1.30	1.32
**Outer regional/remote**		
ICC	0.022	0.029
PCV (%)	na	-32
MOR	1.29	1.35

Notes:
1. ICC, intra-class coefficient; PCV, proportional change in variance (reported change from null model); MOR, median odds ratio.
2. Null model, random intercept only, no covariates; Model 2 adjusted for education, age, sex, country of birth, marital status, self-rated health, chronic disease and physical functional limitation.
3. Wald test that between-area variance for the null model is non zero (i.e. δ_u^2 =0) p<.001. See Appendix G for full details of model diagnostics.

For peer review only

Supplementary table 4. Between-area variation in odds of quality of GP care outcomes

	Long consults		Continuity of care		Care planning	
	**Null model**	**Model 2**	**Null model**	**Model 2**	**Null model**	**Model 2**
**Cities**						
ICC	0.009	0.013	0.007	0.007	0.049	0.039
PCV (%)	na	-44	na	1.5	na	22
MOR	1.18	1.21	1.16	1.16	1.48	1.41
**Inner regional**						
ICC	0.027	0.031	0.017	0.019	0.049	0.048
PCV (%)	na	-16	na	-8	na	4
MOR	1.33	1.36	1.26	1.27	1.48	1.47
**Outer regional**						
ICC	0.033	0.041	0.037	0.037	0.043	0.044
PCV (%)	na	-24	na	-0.4	na	-2.1
MOR	1.37	1.43	1.40	1.40	1.44	1.45

Notes:

1. ICC: intra-class coefficient; PCV: proportional change in variance (reported change from null model), MOR median odds ratio; na, not applicable.
2. Null model, random intercept only, no covariates;Model 2 adjusted for education, age, sex, country of birth, marital status, self-rated health, chronic disease and physical functional limitation.
3. Wald test that between-area variance for the null model is non zero (i.e. δ_u^2 =0) p<.001 for all outcomes.

Supplementary table 5. Between-area variation in the odds of specialist use

	**Null model**	**Model 2**
**Major cities**		
ICC	0.007	0.007
PCV (%)	na	-8
MOR	1.15	1.16
**Inner regional**		
ICC	0.008	0.009
PCV (%)	na	-19
MOR	1.17	1.18
**Outer regional**		
ICC	0.008	0.008
PCV (%)	na	-8
MOR	1.16	1.17

Notes:

1. ICC: intra-class coefficient; PCV: proportional change in variance. Reported change from null model; MOR, median odds ratio; na, not applicable.
2. Null model, random intercept only, no covariates; Model 2 adjusted for education, age, sex, country of birth, marital status, self-rated health, chronic disease and physical functional limitation.
3. Wald test that between-area variance for the null model is non zero (i.e. δ_u^2 =0) p<.001 for major cities and inner regional, <.01 for outer regional/remote. See Appendix G for full details of model diagnostics.

For peer review only

Supplementary table 6. Model 2: fixed effects for quality-of-care outcomes by region

	Cities<br>OR (95%CI)	Inner regional<br>OR (95%CI)	Outer regional/ remote<br>OR (95%CI)
**Long consults**			
University (ref.)	1	1	1
Apprentice/diploma	1.00 (0.97–1.04)	0.95 (0.91–0.99)	0.94 (0.90–0.99)
School certificate	0.96 (0.92–0.99)	0.90 (0.86–0.93)	0.90 (0.85–0.94)
No school certificate	0.96 (0.91–1.01)	0.90 (0.86–0.95)	0.92 (0.87–0.98)
**Continuity of care**			
University (ref.)	1	1	1
Apprentice/diploma	1.07 (1.03-1.11)	1.07 (1.02-1.11)	1.06 (1.01-1.11)
School certificate	1.11 (1.07-1.15)	1.18 (1.14-1.23)	1.18 (1.12-1.24)
No school certificate	1.14 (1.07-1.20)	1.24 (1.18-1.31)	1.15 (1.08-1.22)
**Care planning**			
University (ref.)	1	1	1
Apprentice/diploma	1.21 (1.14-1.29)	1.27 (1.19-1.35)	1.25 (1.15-1.36)
School certificate	1.32 (1.24-1.40)	1.40 (1.31-1.49)	1.43 (1.32-1.55)
No school certificate	1.53 (1.42-1.64)	1.64 (1.52-1.76)	1.61 (1.47-1.77)

Notes:
1. GP, general practitioner; OR, odds ratio; CI, confidence interval.
2. Adjusted for sociodemographic (educational attainment, age, sex, country of birth, marital status) and need (self-rated health status, number of chronic disease, physical functioning) variables.
3. Wald joint test of significance for education p<.001 for all outcomes and regions, except long consults in major cities (p<.05).

Supplementary table 7. Model 2: fixed effects for specialist use by region

**Model 2, specialist use**	Cities<br>OR (95%CI)	Inner regional<br>OR (95%CI)	Outer regional/ remote<br>OR (95%CI)
University (ref.)	1	1	1
Apprentice/diploma	0.94(0.91–0.98)	0.92(0.89–0.96)	0.95(0.91–1.00)
School certificate	0.90(0.86–0.93)	0.91(0.88–0.95)	0.95(0.91–1.00)
No school certificate	0.86(0.81–0.90)	0.85(0.81–0.90)	0.95(0.90–1.01)
p-value for SEP term	<.001	<.001	ns

Notes:
1. OR, odds ratio; CI, confidence interval; SEP, socioeconomic position; ns, not statistically significant.
2. Adjusted for sociodemographic (educational attainment, age, sex, country of birth, marital status) and need (self-rated health status, number of chronic disease, physical functioning) variables.
3. Wald joint test of significance for education cities and inner regional <.001, outer regional not significant.

For peer review only

STROBE Statement—Checklist of items that should be included in reports of ***cohort studies***

	Item No	Recommendation	Page No
**Title and abstract**	1	(*a*) Indicate the study's design with a commonly used term in the title or the abstract	1
		(*b*) Provide in the abstract an informative and balanced summary of what was done and what was found	2
**Introduction**			
Background/rationale	2	Explain the scientific background and rationale for the investigation being reported	4
Objectives	3	State specific objectives, including any prespecified hypotheses	4
**Methods**			
Study design	4	Present key elements of study design early in the paper	5
Setting	5	Describe the setting, locations, and relevant dates, including periods of recruitment, exposure, follow-up, and data collection	5
Participants	6	(*a*) Give the eligibility criteria, and the sources and methods of selection of participants. Describe methods of follow-up	5
		(*b*) For matched studies, give matching criteria and number of exposed and unexposed	na
Variables	7	Clearly define all outcomes, exposures, predictors, potential confounders, and effect modifiers. Give diagnostic criteria, if applicable	6
Data sources/ measurement	8*	For each variable of interest, give sources of data and details of methods of assessment (measurement). Describe comparability of assessment methods if there is more than one group	5 and 6
Bias	9	Describe any efforts to address potential sources of bias	7
Study size	10	Explain how the study size was arrived at	7
Quantitative variables	11	Explain how quantitative variables were handled in the analyses. If applicable, describe which groupings were chosen and why	6-7
Statistical methods	12	(*a*) Describe all statistical methods, including those used to control for confounding	7
		(*b*) Describe any methods used to examine subgroups and interactions	7
		(*c*) Explain how missing data were addressed	6
		(*d*) If applicable, explain how loss to follow-up was addressed	na
		(*e*) Describe any sensitivity analyses	7
**Results**			
Participants	13*	(a) Report numbers of individuals at each stage of study—eg numbers potentially eligible, examined for eligibility, confirmed eligible, included in the study, completing follow-up, and analysed	8
		(b) Give reasons for non-participation at each stage	8
		(c) Consider use of a flow diagram	
Descriptive data	14*	(a) Give characteristics of study participants (eg demographic, clinical, social) and information on exposures and potential confounders	8-9
		(b) Indicate number of participants with missing data for each variable of interest	Table 1
		(c) Summarise follow-up time (eg, average and total amount)	8
Outcome data	15*	Report numbers of outcome events or summary measures over time	Table 1

For peer review only - http://bmjopen.bmj.com/site/about/guidelines.xhtml

Main results	16	(*a*) Give unadjusted estimates and, if applicable, confounder-adjusted estimates and their precision (eg, 95% confidence interval). Make clear which confounders were adjusted for and why they were included<br>(*b*) Report category boundaries when continuous variables were categorized<br>(*c*) If relevant, consider translating estimates of relative risk into absolute risk for a meaningful time period	Figure 2, supplementary tables 3-7<br><br>na
Other analyses	17	Report other analyses done—eg analyses of subgroups and interactions, and sensitivity analyses	10
**Discussion**			
Key results	18	Summarise key results with reference to study objectives	10
Limitations	19	Discuss limitations of the study, taking into account sources of potential bias or imprecision. Discuss both direction and magnitude of any potential bias	12
Interpretation	20	Give a cautious overall interpretation of results considering objectives, limitations, multiplicity of analyses, results from similar studies, and other relevant evidence	10-12
Generalisability	21	Discuss the generalisability (external validity) of the study results	12
**Other information**			
Funding	22	Give the source of funding and the role of the funders for the present study and, if applicable, for the original study on which the present article is based	15

*Give information separately for exposed and unexposed groups.

Note: An Explanation and Elaboration article discusses each checklist item and gives methodological background and published examples of transparent reporting. The STROBE checklist is best used in conjunction with this article (freely available on the Web sites of PLoS Medicine at http://www.plosmedicine.org/, Annals of Internal Medicine at http://www.annals.org/, and Epidemiology at http://www.epidem.com/). Information on the STROBE Initiative is available at http://www.strobe-statement.org.

For peer review only
